# Advanced Carbon Materials Derived from Polybenzoxazines: A Review

**DOI:** 10.3390/polym13213775

**Published:** 2021-10-31

**Authors:** Cecilia Shaer, Leah Oppenheimer, Alice Lin, Hatsuo Ishida

**Affiliations:** 1 Department of Macromolecular Science and Engineering, Case Western Reserve University, Cleveland, OH 44106, USA; cms303@case.edu (C.S.); leo9@case.edu (L.O.); 2 Hathaway Brown School, Shaker Heights, OH 44120, USA; alin@hb.edu

**Keywords:** advanced carbon material, benzoxazine, polybenzoxazine

## Abstract

This comprehensive review article summarizes the key properties and applications of advanced carbonaceous materials obtained from polybenzoxazines. Identification of several thermal degradation products that arose during carbonization allowed for several different mechanisms (both competitive ones and independent ones) of carbonization, while also confirming the thermal stability of benzoxazines. Electrochemical properties of polybenzoxazine-derived carbon materials were also examined, noting particularly high pseudocapacitance and charge stability that would make benzoxazines suitable as electrodes. Carbon materials from benzoxazines are also highly versatile and can be synthesized and prepared in a number of ways including as films, foams, nanofibers, nanospheres, and aerogels/xerogels, some of which provide unique properties. One example of the special properties is that materials can be porous not only as aerogels and xerogels, but as nanofibers with highly tailorable porosity, controlled through various preparation techniques including, but not limited to, the use of surfactants and silica nanoparticles. In addition to the high and tailorable porosity, benzoxazines have several properties that make them good for numerous applications of the carbonized forms, including electrodes, batteries, gas adsorbents, catalysts, shielding materials, and intumescent coatings, among others. Extreme thermal and electrical stability also allows benzoxazines to be used in harsher conditions, such as in aerospace applications.

## 1. Introduction 

Carbon has a wide range of applications and has been used since ancient times as a fuel, adsorbent, additive to iron, lubricant, pencil core, gemstone for accessories, and more recently as carbon fibers. A renaissance of advanced carbon materials from the theoretical prediction of the existence of a spherical C_60_ material that was reported by Osawa in 1970 [1] with the subsequent discovery of the C_60_ material in sooty residue while evaporating carbon under helium by Kroto et al. [2]. It was named Buckminsterfullerene (Buckyball) after the geodesic architecture by an architect, Buckminster Fuller. The discovery of fullerene led to the first Nobel prize in carbon materials, although this material in a terrestrial environment has long been known among astrophysicists. The physical properties of fullerene were fascinating, and many unusual properties, including electrical conductivity, magnetism, and others, have been observed. However, it was of limited use as a material for mechanical applications due to the small aspect ratio of 1.

This all changed when Iijima reported the detailed study of an elongated form of Buckyball, termed carbon nanotube (CNT) [3]. CNT has a comparable diameter to Buckyball but has an elongated shape to have an aspect ratio of hundreds, allowing it to effectively reinforce a weak material such as a bulk polymer, ceramic, or metal matrix. CNT, both with single and multiwalled tubes, has been extensively studied since then.

If the carbon tube is cut open to spread into a two-dimensional sheet, it produces another effective reinforcing material. First reported by Geim and Novoselov, this material is called graphene and was initially produced by peeling off a single sheet from graphite [4]. The discovery of graphene then resulted in the second Nobel prize in the carbonaceous material field. For the application of graphene to composite materials, peeling graphene one by one from graphite is impractical. It is now produced from the chemical exfoliation of graphene sheets in graphite by modifying the structure into graphene oxide (GO), typically by Hummers method [5] and its variation, followed by reducing chemical defects either by chemical means or heat. While the structure is not as ordered as the graphene obtained from peeling graphite, it functions to be an effective reinforcing material, though the electrical conductivity is severely reduced from perfect graphene.

Today, there are a vast number of advanced carbon materials being studied. They include SWCNT, MWCNT, graphene, graphene oxide, carbon-based supercapacitors or electrode materials, metal-free carbon catalysts derived from organic polymers, carbon quantum dots, and carbon aerogels, just to name a few. For more details, readers are referred to monographs and many excellent reviews [6,7,8,9,10,11]. Interests in discovering yet further advanced carbon materials, such as graphane, a fully hydrogenated version of graphene, and graphone, a half hydrogenated graphene [12,13] are strong. One of the common drawbacks of advanced carbon materials is the lack of mass-productive methods. This makes it quite expensive to produce those materials. It is, therefore, of great academic and technological interests to discover materials or develop methodologies that allow mass production of advanced carbonaceous materials. Polybenzoxazine is the latest commercialized new class of polymers that is leading the renaissance of thermoset research. Among many unusual properties and advantages [14,15,16,17,18,19,20], extremely high degree of molecular design flexibility and char formation are two of the most notable advantages for the carbon applications. The current review is a comprehensive description of the fundamental and applied study of benzoxazine resins and cross-linked polybenzoxazines towards carbon material preparation.

## 2. Synthesis of Carbonaceous Materials from Polybenzoxazines (General Procedures)

**Figure 1:**
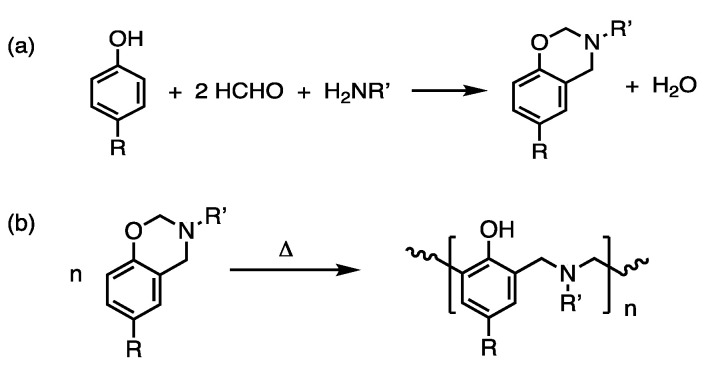
(**a**) Benzoxazine monomer synthesis from a phenolic derivative with the open *o*-positions, a primary amine, and formaldehyde. (**b**) Cationic ring opening polymerization of benzoxazine monomer showing no production of reaction side products.

Polybenzoxazines are typically prepared from a benzoxazine monomer; however, there are also oligomeric precursors, such as main-chain, side-chain, and telechelic type precursors. The structures and properties of the resultant polybenzoxazines from these precursors are different from the ordinary monomeric precursors. For more details of various benzoxazine reins, extensive literature is available [14,15,16,17,18,19,20]. There are a variety of synthetic methods even for monomeric benzoxazines, and one can choose different approaches depending on the possibility of adopted chemistry that might interfere with parts of molecules during the synthesis [21,22]. Polybenzoxazines are usually obtained by heating a benzoxazine monomer to the temperature range of 140–240 °C with or without an added initiator and/or catalyst. Benzoxazine has its own intrinsic ability to undergo cationic ring-opening polymerization [23]. However, to increase the rate of polymerization or reduce the polymerization temperature, one often adds an initiator and/or catalyst, such as phenol, which may come from impurities or benzoxazine oligomers [23]. A very standard benzoxazine monomer synthesis and its polymerization scheme is shown in Figure 1a,b.

Polybenzoxazines offer one of the highest char yields among all processable polymers in the range between 35 and 75% [24]. Therefore, obtaining certain shapes of carbonaceous product can be readily achieved with good yield. Depending on the processing method of forming the initial precursor material and the following carbonization processes, various forms of carbonaceous products, such as solid blocks, carbon aerogels, monoliths, nanofibers, and carbon dots, can be obtained. In this section, representative methods of preparing those products will be described.

### 2.1. Solid Blocks and Composites

As benzoxazine resins polymerizes without reaction side products, preparation of solid blocks should, in theory, be straightforward. However, caution must be taken to dry the solvent used to synthesize the resins and purify. Benzoxazines usually like to hold onto the solvent rather tenaciously and thus the residual solvent can evaporate during polymerization and leads to porous samples unless it is carefully dried. Another potential problem is the evaporation of monomers prior to and during the polymerization. Evaporation of the monomer prior to the polymerization is not as a serious problem as it is during the polymerization. Although not a serious problem, evaporation is reported to take place in particular for small, low viscosity benzoxazine resins [24,25,26]. However, a smart benzoxazine monomer having a AB-type functionality within the same monomer undergoes Diels–Alder reaction shortly before the polymerization reaction to minimize the evaporation problem has also been reported [27]. The advantages of this approach are the low resin viscosity during processing before the Diels–Alder reaction and increased molecular weight to prevent evaporation upon Diels–Alder reaction when the processing temperature increased.

### 2.2. Carbon Aerogels

An aerogel distinguishes itself from a foam by having pore structures all connected, so the internal pore surfaces are also available for adsorption and catalysis. It was first reported by Kistler in 1931 [28]. Since the report in the early 1990s [29], carbon aerogels have been actively studied. Due to the capillary force of the liquid evaporating, the pores of the aerogels tend to collapse. Thus, supercritical drying is typically the choice of drying methods as the surrounding is in the triple states and the effect of the capillary force can be minimized. However, this requirement can result in costly consequences as the supercritical drying device is expensive and has a limited size. Alternative techniques are also available, freeze drying and ambient drying, though they are applicable only to certain polymers due to the requirement of the aerogel strength to resist collapsing during drying.

In the food industry, freeze drying is actively exercised and large facilities are readily available. The morphology of a layered ice matrix can influence the final morphology of the aerogel to be layered, helping to open up the internal space even better. Schiraldi et al. studied the effect of frozen water morphology on the aerogel structure [30,31,32]. 

**Figure 2:**
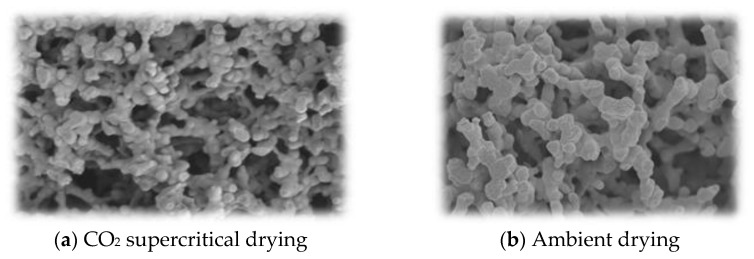
Comparison of polybenzoxazine aerogels prepared by (**a**) supercritical drying and (**b**) ambient drying showing the similarity of the microstructure. (Reproduced from the work in [36] with permission).

A further inexpensive method is ambient drying. This, too, suffers from the potential collapse of the pores due to the capillary force and thus requires polymer gels that are strong. All these are doable with polybenzoxazine gels. In fact, there have been reports of polybenzoxazine aerogel preparation by both ambient drying [33,34] and freeze drying [35,36]. Although supercritical CO_2_ drying was also attempted [35], direct comparison of CO_2_ supercritical drying and ambient drying methods yielded rather close results as shown in Figure 2 as long as a benzoxazine is used as the precursor [36].

The pore diameter and distribution can be tailored using surfactant to control the gel spheres that comprise the aggregates of those nanospheres, eventually leading to aerogel morphology. Thus, being able to control the pore size and its distribution of carbon aerogels by the type of solvent and surfactant used [36] will be extremely useful for the application of these materials as many catalytic and adsorption phenomena are delicately influenced by the amount of available sites and how they are available to adhering chemicals. 

**Figure 3:**
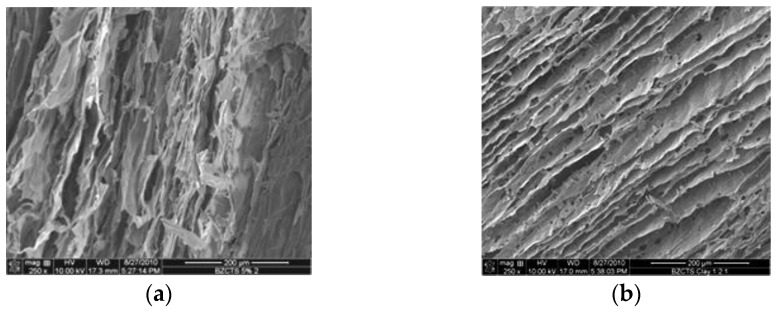
(**a**) Freeze-dried bisphenol A/tetraethylenepentamine (tepa) benzoxazine modified chitosan (1:1) (abbreviated as BA-tepa/CTS). (**b**) Freeze-dried BA-tepa/CTS reinforced with 5wt% of montmorillonite showing improved layer morphology. (Reproduced from the work in [37] with permission).

Freeze drying adds an additional advantage in the pore morphology. As the freezing ice will form a layered morphology [30,31,32], the subsequent aerogel, including its carbonized counterpart, will also form layered morphology as shown in Figure 3 with and without montmorillonite nanoclay used as a reinforcement. The layered morphology is formed by matrix alone with the help of layered ice crystal morphology that acts as a template, while the layered alignment of the aerogel is enhanced by adding a nanoplatelet filler [37]. This allows better availability of the internal surface. By adding nanofiller platelets, this layered morphology can further be enhanced as seen in comparison of freeze-dried polybenzoxazine and freeze-dried and nanofiller-reinforced polybenzoxazine carbon aerogels.

### 2.3. Carbon Nanofibers

Polybenzoxazine-based carbon nanofibers have rarely been reported [38,39]. Si et al. prepared ferromagnetic carbon nanofibers by pyrolyzing electrospun polybenzoxazine nanofibers that are filled with Fe3O4 for water purification application. They blended bisphenol A/aniline-based benzoxazine monomer (abbreviated as BA-a) and poly(vinyl butyral) (PVB) into dimethylformamide (DMF) and tetrahydrofuran (THF) mixed solvent and electrospun the Fe3O4 filler-containing solution. The nanofibers were pyrolyzed under an inert atmosphere at 850 °C.

The electrospun polybenzoxazine nanofibers are prepared in two approaches. As many polybenzoxazine precursors are monomeric, blending with other polymers is done to keep a fibrous form [38,39]. Another approach is to synthesize a main-chain type precursor [40]. This is an oligomeric reactive polymeric chain molecule that can be processed like a thermoplastic and later crosslinked. Therefore, blending with another polymer is not needed to spin the fiber.

**Figure 4:**
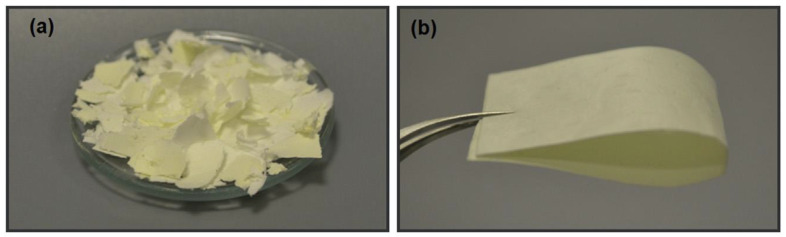
(**a**) A film made of PBA-ad6, and (**b**) electrospun nanowebs from 40% chloroform/DMF mixed solvent solution of PBA-ad6. (Reproduced from the work in [40] with permission).

Ertas and Uyer synthesized a main-chain type polybenzoxazine precursor (abbreviated as PBA-ad6) from bisphenol A and 1,6-diaminohexane in chloroform. The purified product was redissolved in a chloroform/DMF mixed solvent and electrospun. Once the crosslinked polybenzoxazine fibers are made, then pyrolysis using the standard carbonization method will allow carbon fibers or nanofibers to be prepared [40]. A film and nanowebs were prepared using these nanofibers as shown in Figure 4.

### 2.4. Carbon Dots

Carbon nanoparticles show interesting photoluminescent properties in a certain range of size. These are called carbon dots. Preparation of carbon dots from polybenzoxazine has been reported [41,42]. Carbon dots from polybenzoxazine precursors are still in their infancy. Benzoxazine monomer was polymerized catalytically in an autoclave followed by precipitation. The obtained precipitates were then freeze dried to obtain particle precursors. The product was then pyrolyzed in a standard carbonization procedure. Preparation of carbon dots is not unique to polybenzoxazines; however, due to the extremely versatile molecular design flexibility, polybenzoxazine-based carbon dots offer excellent ability to tailor the structures and properties by controlling the chemical composition.

## 3. Fundamental Properties

### 3.1. Mechanism of Carbonization from Polybenzoxazines

**Figure 5:**
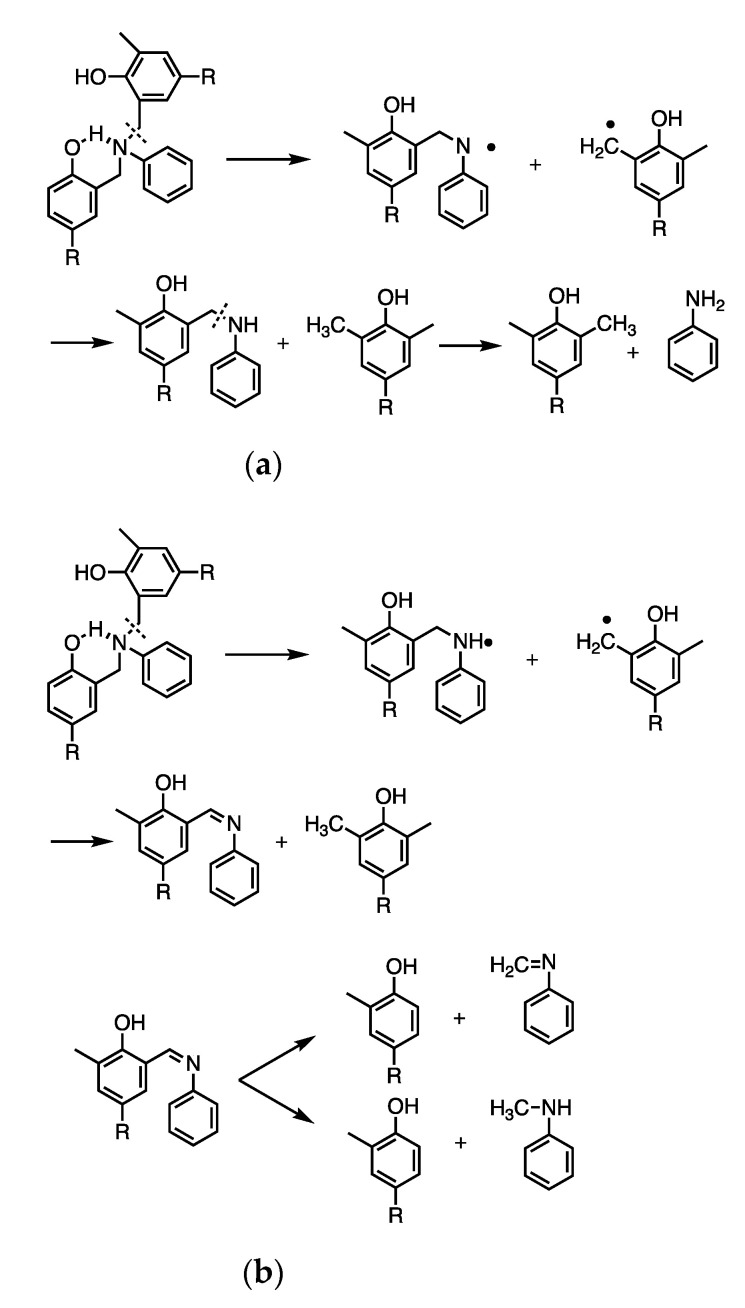
Degradation of hydrogen bonded nitrogen in the Mannich base of aniline-based benzoxazines to produce (**a**) aniline or (**b**) conjugated Schiff bases [45].

**Figure 6:**
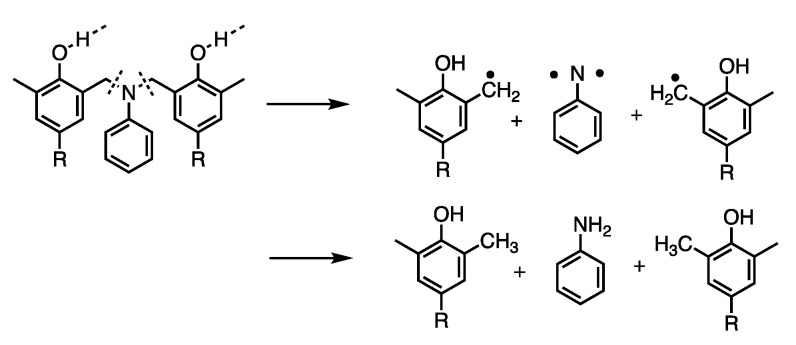
Degradation of non-hydrogen bonded nitrogen in the Mannich base of aniline-based benzoxazines to produce aniline [45].

Carbonization is the decomposition of organic materials to create a carbon residue (the properties and applications of which are heavily discussed here) and is achieved through thermal degradation processes (often pyrolysis) [43]. Degradation and polymerization mechanisms can be derived from identification of thermal degradation products [44,45,46,47,48,49]. Fourier transform infrared spectroscopy (FTIR) and gas chromatography-mass spectrometry (GC–MS) analyses were used in several studies to propose mechanisms of Mannich base cleavage and benzoxazine dimer formation in benzoxazines with various linkages, with a focus on the comparison of aromatic amine-based and aliphatic amine-based benzoxazines, despite the initial degradation products being the same regardless of the phenolic linkages [44]. Low and Ishida found the evolution of ammonia as a degradation product from Mannich base cleavage in aliphatic amine-based benzoxazines, while there was very little evaporation of aromatic amines in aniline-based benzoxazines, as the aromatic units are able to be anchored by polymerized 3-aminophenylacetylene [44,45]. This study also identified NH and substituted benzoxazines or aniline and unsaturated hydrocarbons from further degradation after the Mannich base cleavage of aniline-based benzoxazines, with the route of degradation being dependent on temperature [44]. Another study by Low and Ishida found that *p*-aminotoluene was detected in non-aliphatic amine-based benzoxazines at high temperatures, which indicates the lower thermal stability in benzoxazines with many dangling groups, including the aliphatic amine-based benzoxazines [45]. Degradation products of either aniline or Schiff bases from the cleavage of the Mannich base also yielded two possible degradation processes, seen in Figure 5 and Figure 6, which occur based on whether the nitrogen in the Mannich base is hydrogen bonded [45].

The mechanism of the Mannich base cleavage was also examined. The mechanisms shown in Figure 5 and Figure 6 occurred competitively, as either C–N bond was equally likely to be cleaved [45]. However, Hemvichan et al. identified competitive degradation mechanisms between cleavage of the C–C or C–N bond [49]. In *p*-cresol-based and 2,4-dimethylphenol-based benzoxazines, it was found that, based on the degradation products, small amine linkages were more likely to undergo the C–C cleavage, while large amines underwent the C–N cleavage [49].

**Figure 7:**
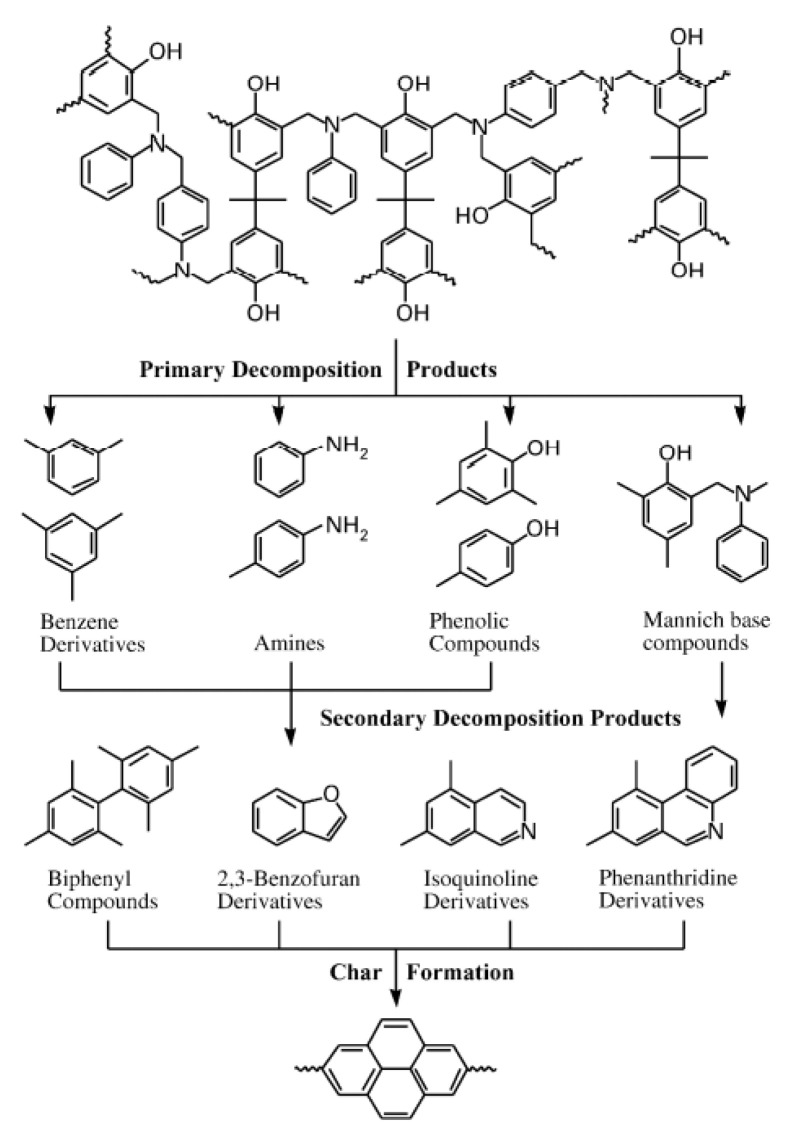
Deamination from C–N cleavage and deaminomethylation from C–C cleavage during char formation from BA-a. [46].

This study also modified *p*-cresol-based benzoxazines with benzoyl chloride and saw esters and secondary amines in the degradation products, as opposed to the primary amines derived from the degradation of *p*-cresol-based and 2,4-dimethylphenol-based benzoxazines [49]. This indicated that for modified *p*-cresol-based benzoxazines, the C–O bond may be cleaved as well due to the resonance stabilization that occurred [49]. A third study by Hemvichan and Ishida confirmed the effects of steric hindrance on bond cleavage and found that in PH-a, BA-a, and BA-35x (synthesized from either phenol, bisphenol A, or 3,5-xylidine, respectively), breaking the C–C bond led to further deaminomethylation, while breaking the C–N bond led to deamination, as shown in Figure 7 [46]. It was also concluded that the R group at the nitrogen atom is significant in determining which competitive mechanism takes place [46].

It was also found that radicals from the degradation of Mannich bases were able to recombine and form dimers [46,49]. In the 2,4-dimethylphenol-based benzoxazines, 2,4-dimethylphenols and tertiary amines were observed to react with hydrogen radicals and reform the benzoxazine monomer in mechanisms where the C–C bond was cleaved [49]. The cleavage of the C–N bond yielded 2,4,6-trimethylphenols, which combine with each other or 2,4-dimethylphenols to form bisphenol compounds [49]. In *p*-cresol-based benzoxazines, biphenyls were also formed through the combination of phenolic radicals that were degradation products [49]. Hemvichan and Ishida also saw the formation of biphenyl compounds that were the result of either the recombination of monomers or were identified as the pyrolysis degradation products [46]. This alternative formation mechanism was proposed due to the lack of biphenyls and other secondary decomposition products being identified in polybenzoxazine networks using mass spectrometry (MS) analysis [46].

The degree of crosslinking in benzoxazines before and after thermal degradation was examined through the formation of char. As char forms as a result of high crosslinking in polymers, the amount of char measured by conducting thermogravimetric analysis (TGA) allows for the proposition of degradation mechanisms that involve crosslinking [44,46,48,49]. In a study by Low and Ishida, an onset of weight loss of BP-apa, HQ-apa, and BZ-apa (synthesized from 3-aminophenylacetate, paraformaldehyde, and either 2,2’-bisphenol, hydroquinone, or 4,4’-dihydroxybenzophenone, respectively) at the same temperatures showed the effect of acetylene groups acting as chain extenders to promote crosslinking [44]. This study also concluded that the phenomenon of crosslinking promoting carbonization is enough to overcome the different phenolic linkages in each of the three benzoxazines, as they yielded similar degradation results [44]. TGA thermograms also support the formation of phenol and bisphenol degradation products released from *p*-cresol-based benzoxazines, as these dimers had lower char yields than the 2,4-dimethylphenol-based and modified *p*-cresol-based benzoxazines since the (unreleased) phenol, bisphenol, and biphenyl units form crosslinks easily (and would thus result in higher char) [46,49]. Bagherifam et al. also found that for polymers formed by the coupling of -NCH_2_ groups on the oxazine ring in poly(PH-ma) (where PH stands for phenol and ma is for methylamine), crosslinking occurred during the degradation after the loss of poly(PH-ma) side chains [48].

Char formation was also studied on benzoxazines with sulfur linkages. Bisphenol-S (abbreviated as BS) and either methylamine- or aniline-based benzoxazines were synthesized with formaldehyde by Liu et al. to form poly(BA-ma) and poly(BS-a). [50,51]. Thermogravimetric mass spectrometry (TG-MS) analysis on poly(BS-a) indicated the detachment of the Schiff base first during thermal degradation, and evaporation of sulfurous gases at higher temperatures of 330 °C caused by the scission of the C–S bond, with more carbocation fragments evolving at higher temperatures [50]. Liu et al. conducted a similar analysis with PBS-m and observed that the methylamine contained weaker C–H and C–N bonds compared to the C–C bonds in the aniline which allowed for the evolution of C and H in degradation products [51]. Hamerton et al. cited that the reason for the scission of the C–N bonds was due to the lower dissociation energy, confirming the results of Liu et al. that sulfur linkages are what contributes greatly to char formation by scission of the C–S bond [51,52]. Zhu et al. also examined the effect of introducing a nitro group to induce polymerization. Due to the strong electron withdrawing abilities of the nitro group, the bonds in the aromatic ring were weaker, causing a higher weight loss for benzoxazines containing the nitro group and the resulting char structure of benzoxazines containing a nitro group was also found to be more chemically stable in various environments [53].

Coupling and degradation products can be further used to indicate ring opening polymerization mechanisms after carbonization. Bagherifam et al. observed that dimers formed by coupling could undergo further polymerization from the alkyl amine and diamine linkages formed during the polymerization process [48]. Low and Ishida also examined the mechanisms during the polymerization process, and an FTIR analysis found that thermally stable carbonyl structures were formed [44]. Both ring-opening polymerization and the formation of carbonyl groups were aided by the presence of metal salts in a study conducted by washing bisphenol A benzoxazine with Cu salts, though after the initiation step, the presence of the salts did not catalyze the benzoxazine polymerization [47].

The effects of the presence of metal salts on the functional group development was also observed in addition to effects on polymerization. Low and Ishida found an increase of carbonyl formation (indicated by FTIR) in benzoxazines that had been washed with metal salts due to the oxidative properties of the transition metals used in the salts [47]. Zhu and Gu looked at the ability of nitrogen in Mannich bridges to form coordination interactions with transition metals by using La_2_O_3_. The coordination allowed the La_2_O_3_ to both anchor the aniline in degradation and raise the molecular weight of the benzoxazine sample, leading to an increase in char formation [54].

**Figure 8:**
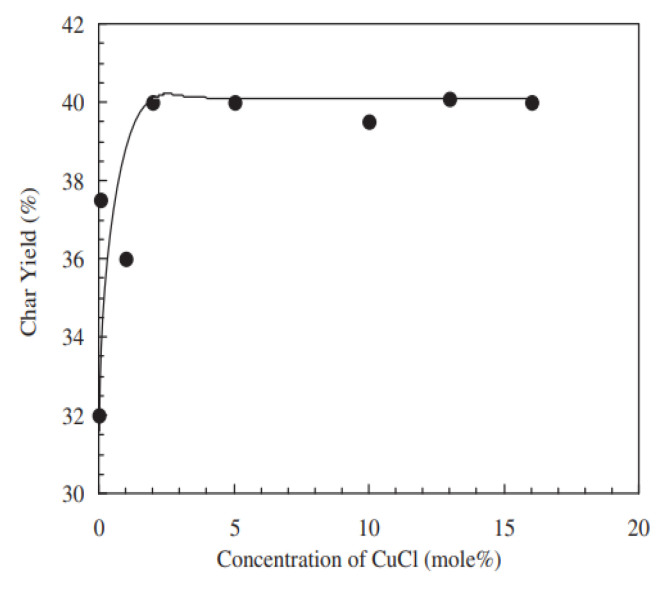
Effect of CuCl initiator concentration on char yield for 3-aminphenylacetylene based benzoxazines (Reproduced from [47] with permission).

Hamerton et al. also refined a method to predict the char yields for polybenzoxazines under thermal degradation using quantitative structure property relationships (QSPR) models [55]. QSPR models were found to be effective for aniline- and bisphenol-A-based benzoxazines, as it had high linear regression (R_2_) values of greater than 0.99 when compared with experimental results from TGA thermograms, and accurately matched the degradation patterns derived from earlier experiments [55]. The QSPR model found that benzoxazines containing 3-aminphenylacetylene, which induce crosslinking, would yield high amounts of char [55]. The char yields for relative amounts of transition metal initiators were also measured, which demonstrated that more initiator yielded more char up to 5 mol%, as shown by Figure 8 [55]. Earlier studies, such as by Low and Ishida, also examined the effect of the presence of transition metal salts on char yield and found that the impact was insignificant on BA-a, unlike what Hamerton et al. predicted for the same compound [47,55].

### 3.2. Electrochemical Properties

**Figure 9:**
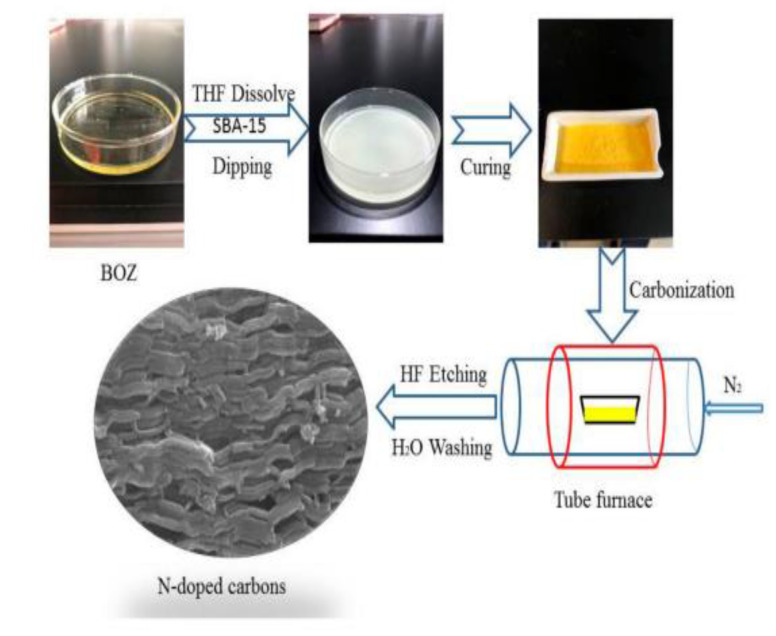
Preparation process of nitrogen-doped carbonized benzoxazine. (Reproduced from the work in [57] with permission).

Benzoxazine-derived porous carbon material, particularly when doped with heteroatoms, can exhibit pseudocapacitance due to their porosity, in addition to high electrical conductivity. Gao et al. examined the electrochemical properties of carbon aerogels derived from nitrogen and sulfur co-doped polybenzoxazines and observed that the introduction of sulfur was key in preventing pore shrinkage, in addition to producing electrically active sites through redox reactions with the nitrogen and sulfur created pseudocapacitance (which was also observed in the nitrogen-only-doped benzoxazine) as a result of the enhanced porosity [56]. Similarly, Zhang et al. observed similar properties in nitrogen doped benzoxazines (prepared as shown in Figure 9), finding that the sample with a lower nitrogen content had both a larger specific surface area and larger pore volumes than others [57]. Pores with higher surface areas have an increased ability to accumulate charges and transport ions, thus porosity measurements are crucial to understanding the electrochemical properties of benzoxazines [56,57]. Aside from porosity in general, mesopores can create ion transport channels within the carbon material (and grant ions entry to the electrode), thus highly mesoporous benzoxazines are highly suitable as electrodes [56].

**Figure 10:**
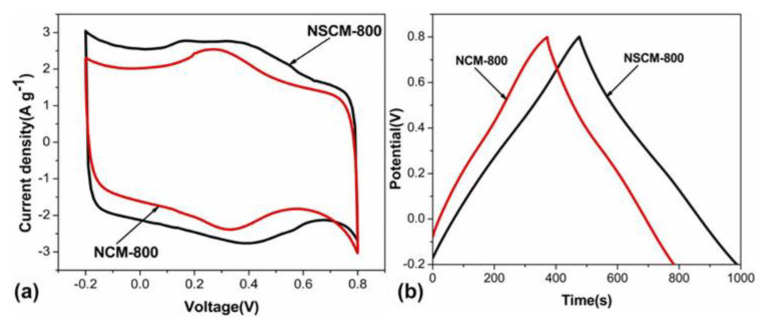
CV curves (**a**) and GCD plot (**b**) of nitrogen and sulfur co-doped benzoxazine at various scan rates (**a**) or current densities (**b**). (Reproduced from the work in [56] with permission).

These electrochemical properties are confirmed with cyclic voltammetry (CV) and galvanostatic charge–discharge (GCD) testing. The shape of CV curves for a nitrogen- and sulfur-doped benzoxazine sample seen in Figure 10a indicates a high capacitance and ability to be charged and discharged rapidly, which gives these benzoxazines ideal double-layer capacitor (EDLC) behavior [56,57]. CV curves of this sample, as well as a nitrogen-only-doped sample, also highlight the pseudocapacitance of the benzoxazine, which is thought to originate from redox reactions with the heteroatoms [56]. The large area of the CV curve seen in Figure 10 indicates both good performance at various different scan rates, and the ability to reversibly absorb–desorb free ions [57].

GDC curves can be used to determine further capacitive and current properties. From the discharge time indicated on the GDC curve in Figure 10, Gao et al. observed a higher specific capacitance in sulfur doped benzoxazines, proposed to be due to the co-doping of nitrogen and sulfur [56]. When observed at different current densities, the isosceles triangular shape indicates the gradual disappearance of pseudocapacitance and maintenance and thus good electrochemical performance [56]. This phenomenon was also observed by Zhang et al., who demonstrated that nitrogen-doped samples had excellent cycling stability, especially in comparison to activated samples [57]. This led to the conclusion that heteroatom doping, with nitrogen in particular, enhances electrochemical performance of benzoxazines.

## 4. Applications

### 4.1. Topology of Polybenzoxazine-Based Carbon 

#### 4.1.1. Carbon Films

**Figure 11:**
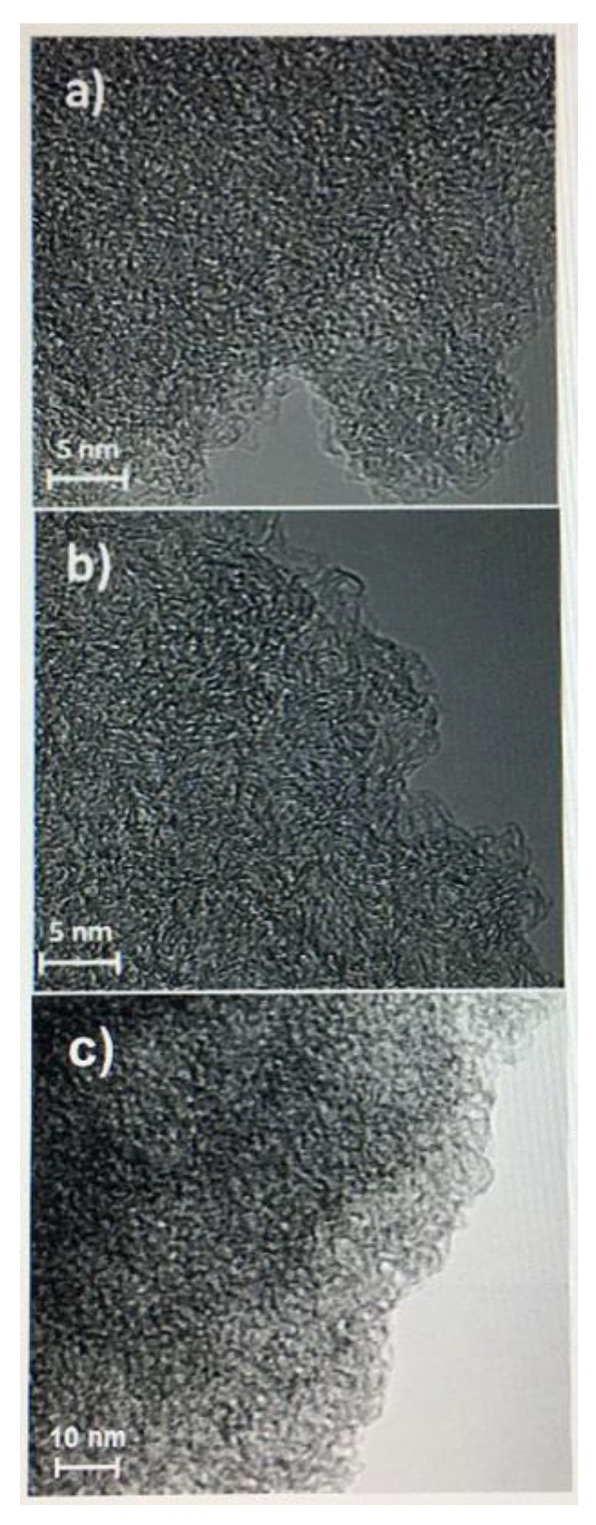
TEM images of carbonized benzoxazine films that are (**a**) BA-a- or (**b**) PH-ddm-based and (**c**) carbonized PI film (reproduced from the work in [58] with permission).

Benzoxazines can also be prepared as films, as seen in Figure 11, which are similar to films made from easily carbonized polyimides (PI) or other materials [58]. Takahashi et al. prepared carbon films derived from either bisphenol A/aniline (abbreviated as BA-a) or phenol/diaminodiphenylmethane (abbreviated as PH-ddm) and compared with the polyimide made from pyromellitic dianhydride (PD) and oxydianiline. These films are characterized by X-ray diffraction (XRD) and X-ray photoelectron spectroscopy (XPS). The XRD results demonstrated that graphitization increased with higher carbonization temperatures, as noted by the higher (002) diffraction peaks [58]. XPS analysis was able to provide similar results, and also demonstrated that despite PI being known as a good graphite precursor, the PH-ddm yielded more significant graphitization, thus further demonstrating the thermal stability of benzoxazine films and that graphitization is possible [58]. 

#### 4.1.2. Polybenzoxazine-Based Carbon Foam

**Figure 12:**
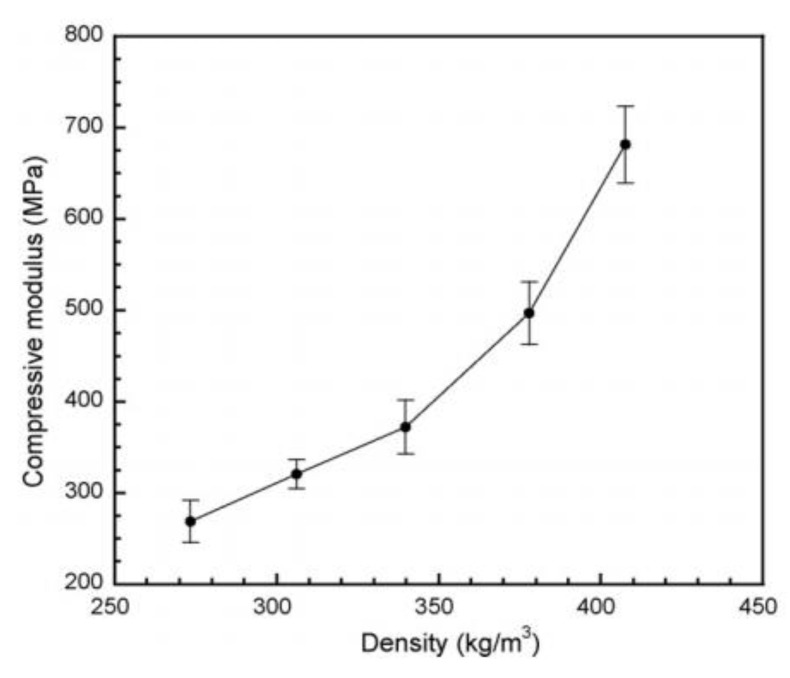
Compression modulus effect with increasing foam density (reproduced from the work in [59] with permission).

**Figure 13:**
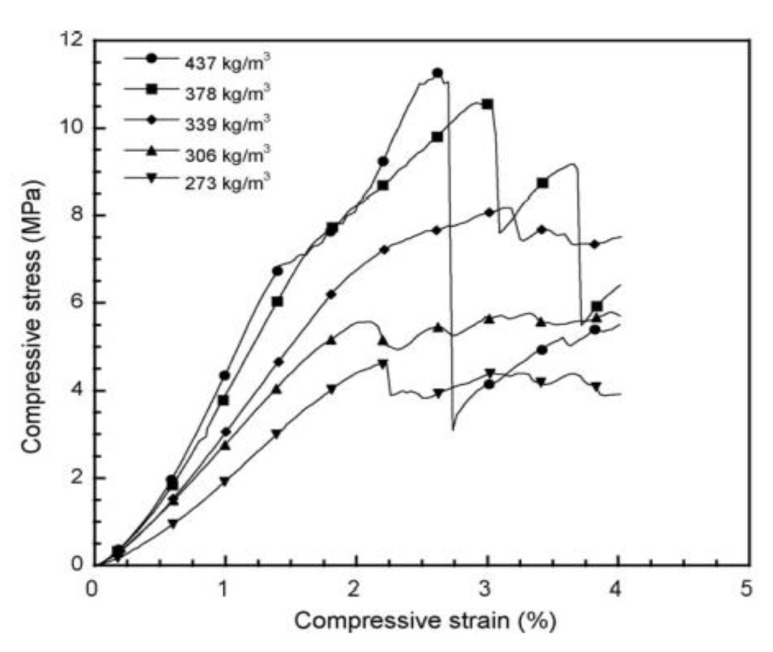
Compressive stress–strain curve of benzoxazine foam at varying densities (reproduced from the work in [59] with permission).

Mechanically and thermally sound polybenzoxazine foams use a variety of methods including the use of foaming agents followed by carbonization. These polybenzoxazine foams released degradation products consisting of benzene derivatives, amines, phenolic compounds, and Mannich base compounds, whereas the azodicarbonamide (AZD) foaming agent released smaller products including CO, CO_2_, N_2_, and NH_3_ [59]. Using a foaming agent alone had no effect on the degradation onset of benzoxazines, though once activated (which can be done by the benzoxazines themselves), the presence of AZD lowered the degradation temperature of benzoxazine [59]. Presence of a foaming agent such as AZD also affected the mechanical properties significantly. As AZD was added, the density of the foam decreased, which in turn reduced the compressive modulus, as shown in Figure 12 [59]. Using AZD in lower amounts to prepare the foams was also found to be more effective in maintaining the compressive strength compared to other techniques, such as the commonly used resin/glass microballoon composite fabrication for benzoxazines used in advanced mechanical applications [59]. In addition to their higher compressive modulus, less dense foams are less likely to fail completely after initial failures than denser foams, thus deforming more flexibly as seen in Figure 13 [59]. The deformation behavior is also attributed to the cell structure of the foam, shown in Figure 13, where foams with thicker cell walls were stronger than those with thinner walls [59]. 

**Figure 14:**
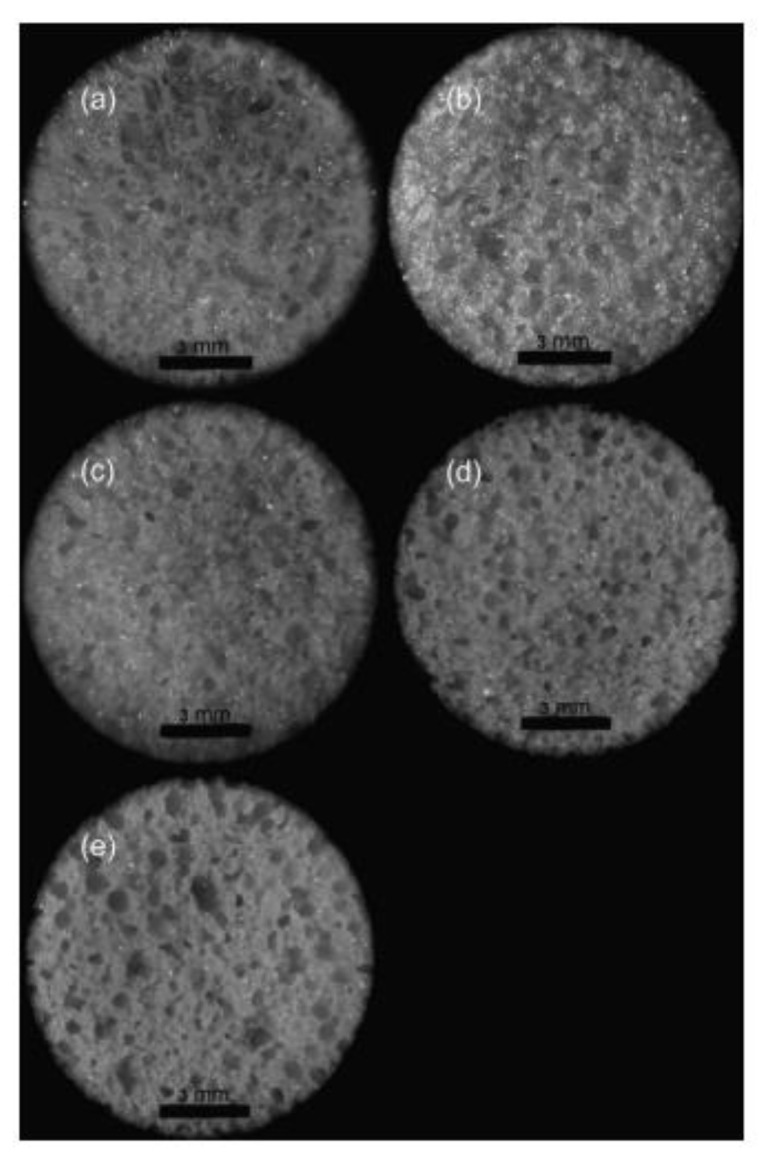
Optical microscope images (with 5 times magnification) of benzoxazine foam at varying densities of (**a**) 407 kg/m^3^, (**b**) 378 kg/m^3^, (**c**) 339 kg/m^3^, (**d**) 306 kg/m^3^, and (**e**) 273 kg/m^3.^ (reproduced from the work in [59] with permission).

Optical microscopic images of the foams with varying density are shown in Figure 14 [59]. Although the void size appears large, the images are magnified 5 times of the actual sample. 

**Figure 15:**
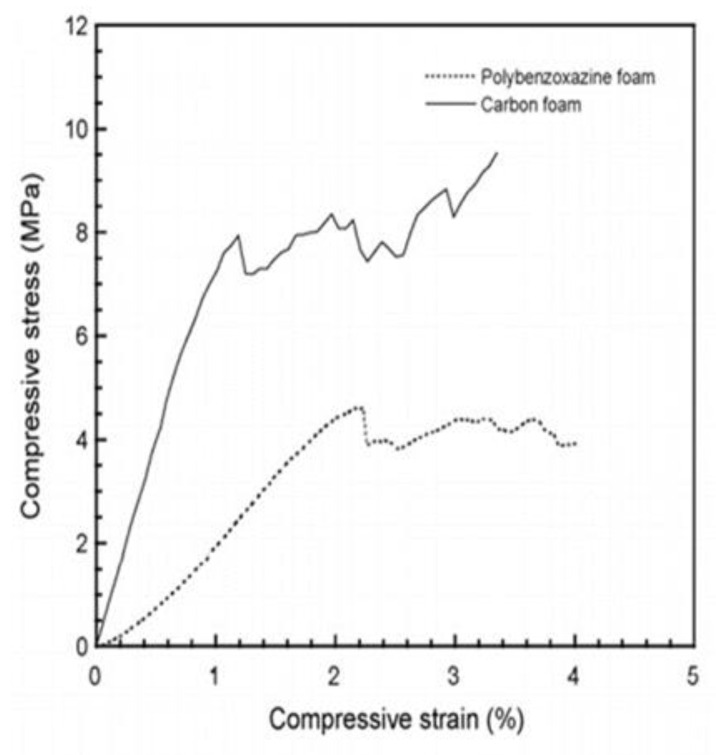
Compressive stress–strain curve of polybenzoxazine foam and carbon foam (reproduced from the work in [59] with permission).

As seen in Figure 15, both the compressive strength (9.5 MPa) and compressive modulus (829 MPa) in the carbonized foam were significantly higher than the pre-carbonized foam, a trend which holds true even after normalization of the properties based on the foam density.

#### 4.1.3. Carbon Nanofibers

**Figure 16:**
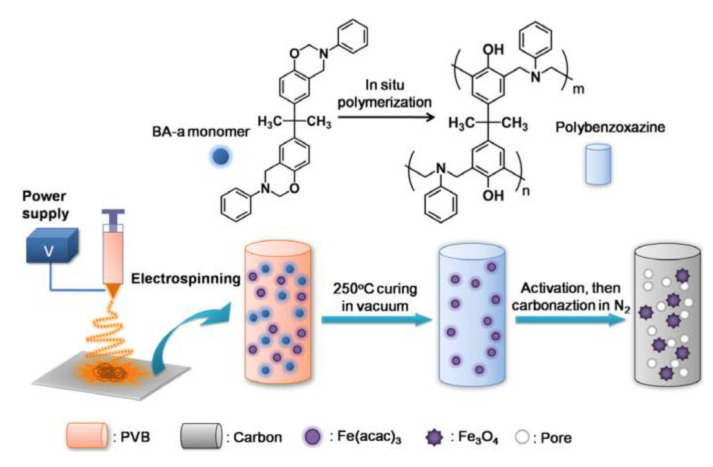
Formation of A-Fe@CNF through BA-a polymerization, electrospinning, curing, activation, and carbonization (reproduced from the work in [39] with permission).

Carbon nanofibers (CNFs) can be derived from polybenzoxazines through several manufacturing processes. Solid and porous CNFs are commonly manufactured using electrospinning, which is helpful for the addition of inorganic nanocrystals. While electrospinning is typically the base process of CNF manufacturing, porous CNFs require additional processes, such as activation in KOH solutions, along with other considerations (to be discussed later in Section 4.2.2 along with the effects of porosity) [60]. Electrospinning was used by both Ren et al. and Si et al. to create Fe_3_O_4_ nanocrystals from a precursor material, Fe(acac)_3_ through oxidation, that was then mixed into the polybenzoxazine nanofibers [39,61]. When considering porous CNFs as well, the electrospinning can be very beneficial, as this process leads to the decomposition of KOH solutions and subsequent release of CO and H_2_O gases formed during carbonization, which in turn created a porous structure [61]. Figure 16 highlights this process, noting the activation step used for porous CNF production. Si et al. also studied the effects of polyvinyl butyral (PVB) precursor activation coupled with electrospinning and observed higher crystallinity in the Fe_3_O_4_ nanocrystals in environments that contained lower proportions of the PVB precursor (and higher proportions of BA-a instead), as the poly(BA-a) provided a more stable matrix to provide stability for the ferrous nanocrystals to aggregate externally [39]. This BA-a doping in the formation of benzoxazine-based CNFs was also observed by Zhu et al., which used a ring-opening crosslinking reaction for BA-a to prevent linkages of the precursor fibers [62].

**Figure 17:**
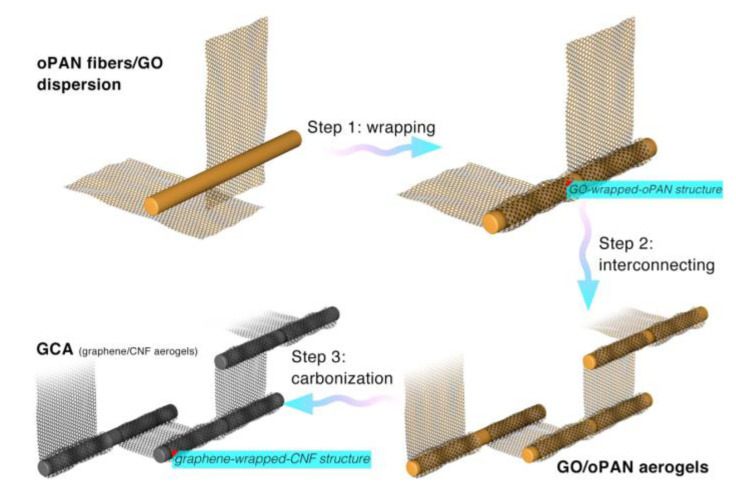
Formation process of GCAs through wrapping, interconnecting, and carbonization (reproduced from the work in [63] with permission).

The mechanisms of graphene formation and manufacturing for CNFs in the presence of Fe and Co were also observed by Zhu et al. and Huang et al. It was found that the presence of these metals improved the graphitization from carbonization, confirmed from XRD [62]. In addition to graphene, metallic nanocrystals formed from the combination of Co(acac)_3_ and Fe(acac)_3_, similar to the post-carbonization formation of Fe_3_O_4_ nanocrystals observed in previous studies [62]. Graphene oxide wrapped nanofibers were also able to be synthesized by Huang et al. using the process illustrated in Figure 17 [63]. When the resulting wrapped fiber network is carbonized, graphene graphene composite aerogels (GCAs), can be formed [63]. 

#### 4.1.4. Carbon Nanospheres/Nanodots

**Figure 18:**
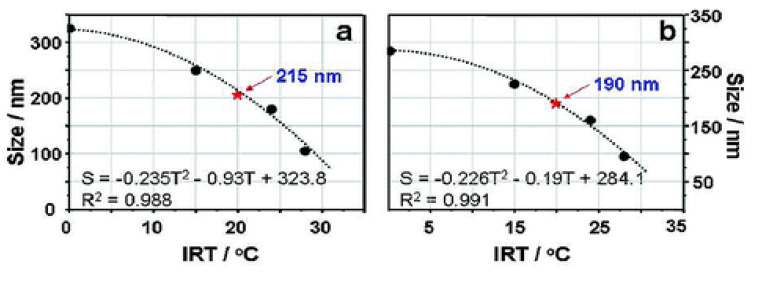
Nanosphere size as it relates to IRT for nanospheres before (**a**) and after (**b**) carbonization (reproduced from the work in [64] with permission).

Several processes to form carbon nanospheres have been observed based on the fundamentals of benzoxazine chemistry, which were found to have good porosity control [64,65]. Wang et al. considered a synthesis that involved the formation of carbon nanospheres from carbonization of benzoxazine spheres, and that controlling the initial reaction temperature (IRT), and thus the reaction rate, affects the size of the resulting nanospheres (where higher IRTs yielded smaller nanospheres), as shown in Figure 18. Zhao et al. also developed a novel nanosphere formation mechanism, which polymerized aggregated benzoxazine from 3-aminophenol and formaldehyde, yielding uniform polymer spheres (APFS) and found a relationship between the IRT and the uniformity of the spheres, where slower reactions (lower IRT) resulted in less uniform APFS [65]. At an IRT of 0 °C, the APFS were not very uniform, while order of the nanospheres increased at higher IRTs, particularly in the range of 10 °C to 60 °C [65]. 

The IRT, however, was not the only factor that determined APFS size. Monomer and reagent concentration also play a significant role, where increased concentrations of 3-aminophenol led to the formation of larger nanospheres [65]. Zhao et al. also noticed that when the nanosphere droplet size increased at higher monomer concentrations, there were more precursor particles inside the spheres, so the sphere volume was further increased (which was also aided by the subsequent decrease of solvent concentration) [66]. In addition to altering the sphere size, Zhao et al. found that monomer concentration affected the size and morphology of the pores within the nanosphere [66]. Dai et al. further examined the pore properties of N-doped carbon@mesosilica hollow spheres (N_x_C@mSiO_2_) and found a hollow cavity structure and unique stable amphiphilic properties [67]. 

**Figure 19:**
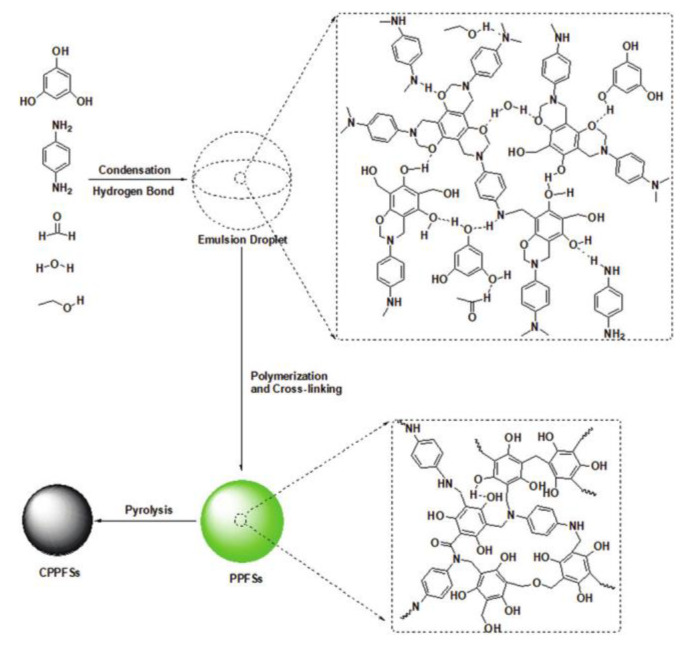
Catalyzed ring opening polymerization and carbonizations to form carbon nanospheres (reproduced from the work in [66] with permission).

The role of catalysts and carbonization in nanosphere formation was also studied, where elements of the nanosphere were sometimes found to have their own catalytic capabilities, in addition to affecting the behavior of external catalysts. Zhao et al. found that multiple amine functionality of benzoxazine-reagent *p*-phenylenediamine allowed it to catalyze the polymerization of formaldehyde and resorcinol while also participating in the reaction prior to the ring-opening polymerization, illustrated in Figure 19 [66]. Du et al. further showed that ethylenediamine (EDA), when used as a reagent, was also found to both participate in the polymerization and have a catalytic effect [68]. N_x_C@mSiO_2_ was also studied as a catalyst with platinum, along with plain nitrogen-doped carbon (N_x_C), and it was found that its amphiphilicity significantly affected its catalytic properties by allowing for increased water adsorption along with the desorption of byproduct water from the reagent’s active sites [67]. In addition to the amphiphilicity, the doping of nitrogen was further beneficial in catalytic functions in comparison to undoped carbon [67]. In addition to the amphiphilicity, the doping of nitrogen was further beneficial in catalyst functions (in comparison to undoped carbon) [67]. Several studies also demonstrated an environmentally friendly method of nanosphere formation, using “green” solvents, such as water or a solventless system, and lack of a catalyst, that could be used in larger production scales [64,65,66,67].

Carbon nanospheres also have several beneficial properties, including gas adsorption (to be discussed in greater detail in Section 4.2 and Section 4.1) and good electrochemical performance, indicating their promising applications as supercapacitors [66,68]. CV analyses demonstrate pseudocapacitive properties of carbon nanospheres doped with heteroatoms [66]. In nanospheres with reduced amounts of heteroatoms, pseudocapacitance and other electrochemical properties were enhanced [66]. Du et al. attributed this to the thin shell and high surface area of carbon nanosphere particles, and a mesoporous structure that allowed for easy electrolyte flow [68]. In addition to their valuable electrochemical properties, carbon nanospheres have high thermal properties, especially after graphitization. Zhu et al. also looked at the thermal conductivity of benzoxazine graphitic carbon nanoparticles (GCPs) and observed a relationship between graphitization and thermal conductivity in addition to carbonization temperature [69]. Graphitization was also found to influence electrochemical storage capacity in carbon nanospheres [68].

### 4.2. Polybenzoxazine-Based Carbon Aerogels

#### 4.2.1. Controlled Microporosity and Mesoporosity

Carbonized benzoxazines can be formed into carbon aerogels by removing the liquid phase of the material, thus creating a porous structure. As a result of this aeration, three classes of pores defined by IUPAC can be formed including micropores (with diameters less than 2 nm), mesopores (with diameters between 2 nm and 50 nm), and macropores (with diameters greater than 50 nm), each with different adsorption and transport capabilities [70]. Pores are often defined by their morphology and a number of size characteristics including pore volume, pore diameter, Brunauer–Emmett–Teller (BET) surface area (a method of calculating pore surface area by measuring monolayer coverage of gases, such as N_2_, at various temperatures and pressures), and pore distribution (the relative amount of micro, meso, and macropores in a sample) [71]. 

**Figure 20:**
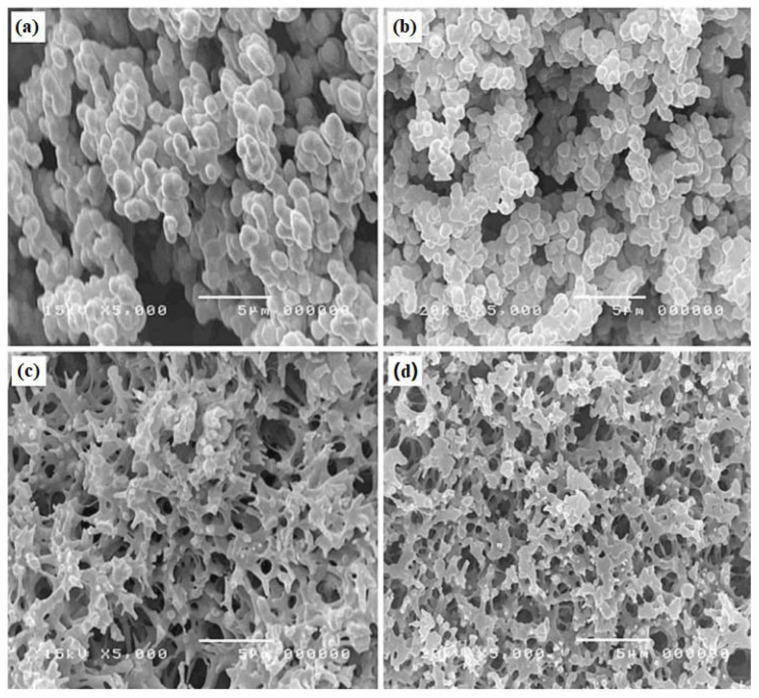
SEM images of carbon aerogels at (**a**) 20 wt% monomer (cured), (**b**) 20 wt% monomer (uncured), (**c**) 40 wt% monomer (cured), and (**d**) 40 wt% monomer (uncured) (reproduced from the work in [33] with permission).

Such characteristics are found to be highly tailorable by a number of factors such as the type and amount of monomer, solvent type, bulk density, use of surfactants or nanoparticles, or other processes including carbonization. The effects of monomer concentration on morphology and pore dimensions in carbon aerogels were observed. Increasing the monomer concentration has been found to have a definite, but inconsistent, effect on pore structure. Lorjai et al. found an inverse relationship between porosity and monomer concentration, where upon increasing monomer concentration, the morphology of poly(BA-a) aerogels shifted from agglomerated carbon particles to a smooth polymer network with an open microporous structure, as seen in Figure 20 [33]. Thubsuang et al. also reported a change in morphology upon increasing benzoxazine monomer concentration, where a fused structure was observed at 45 wt% benzoxazine, while at lower concentrations (25 and 35 wt%), a 3D interconnected structure was seen [34]. In addition to affecting the pore structure, monomer concentration has been found to affect the pore dimensions and other characteristics (such as BET surface area, bulk density, and pore volume), generally in a monotonic pattern [33,34,35]. Lorjai et al. found an increase in only BET surface area of micropores (from 296 m^2^/g to 317 m^2^/g) by increasing the monomer concentration in carbon aerogels from 20 wt% to 40 wt% [33]. Mesopores, on the other hand, were found to decrease in BET surface area upon this change (from 103 m^2^/g to 84 m^2^/g), which is similar to the trend Mahadik-Khanolkar et al. observed with micropore BET surface area upon increasing monomer concentration (in the range of 5 wt% to 20 wt%) [33,35]. Mahadik-Khanolkar et al. also observed a monotonic increase in bulk density and a general increase in total pore volume, and pore diameter (for micro and mesopores) upon increasing the benzoxazine monomer concentration [35]. 

While the aforementioned studies mostly found monotonic correlations between monomer concentration and pore characteristics, there have been some deviations from this trend. Thubsuang et al. observed that the benzoxazine with the middle value of monomer concentration (35 wt%) exhibited the highest BET surface area and mesopore volume [34]. In this study, they also found that the micropore and total volume both decreased monotonically with increasing monomer concentration, which, despite showing a clear relationship between the two variables, disagrees with the trend observed by Mahadik-Khanolkar et al. [35]. These studies demonstrate that while monomer concentration clearly does influence porosity, there is no consistent pattern that makes monomer concentration a predictable alteration, possibly due to the influence of other factors that require further examination. 

In addition to the amount of reagent or monomer used, the structure of the monomer, which can be tailored by altering the phenolic and amine components, itself is key in developing controlled porosity due to the intermolecular bonding that takes place. Zhang et al. used a variety of amines in the benzoxazine monomer synthesis and observed that the length of the carbon chain and amine functionality had a significant influence on the pore distribution [72]. They found that primary amines, as opposed to diamines, and amines bonded to longer carbon chains resulted in wider distributions, though all samples were still predominantly microporous [72]. On the other hand, bimodal or trimodal distributions were found from benzoxazines synthesized from larger amines, which is all due to the weaker hydrogen bonding in primary amines with longer alkyl chains [72]. Abuzeid et al. also examined the effects of nitrogen doping and found that it dramatically increased BET and micropore surface areas (N-doped benzoxazine had a BET and micropore surface area of 1469 m^2^/g and 989 m^2^/g, respectively, while the highest of these measurement in undoped carbon were 86.4 m^2^/g and 12.58 m^2^/g, respectively) [42,73]. These studies emphasize the importance of hydrogen bonding in porosity, and that chemical structures which affect hydrogen bonding (amine structure and heteroatom doping) should thus be highly considered factors. 

**Figure 21:**
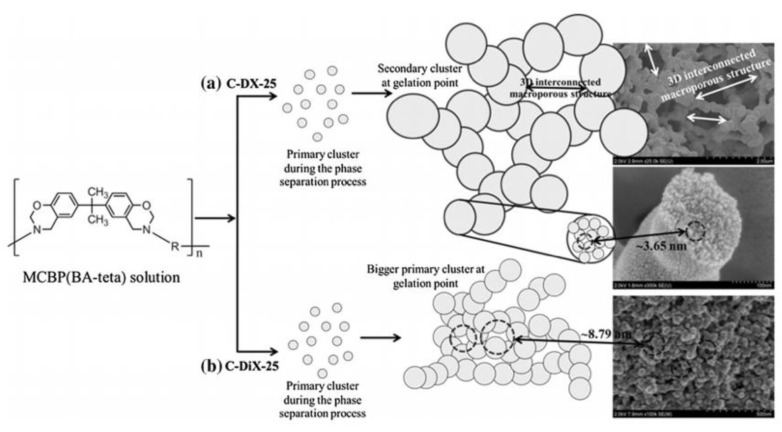
Phase separation of MCBP(BA-teta) solution to form secondary (**a**) and large primary (**b**) clusters and resulting scanning electron microscope (SEM) images of their morphology (reproduced from the work in [34] with permission).

Other factors can affect pore qualities in a clearer way, such as solvent choice, where unfavorable interactions between the monomer and solvent would yield aggregated primary (and thus secondary) clusters to form an interconnected porous structure, shown in Figure 21 [34]. 

The distribution of micro, meso, and macropores can also be tailored through the bulk density, where denser aerogels were primarily more microporous and mesoporous (following the expected trend of the higher surface area to pore volume ratios that come with smaller pores resulting in denser materials) [34,74]. Mahadik-Khanolkar et al. noticed that altering bulk density could be used to tailor the relative pore volume ratios. They also noticed a dramatic change by increasing the bulk density, where the relative number of micropores (less than 300 nm in size) dramatically increased from 1.4–4% to 75–85% [35]. Thubsuang et al. found a similar correlation where the relative micropore volume increased from 6.0% to 17.9% as bulk density increased over 3-fold (from 0.149 g/cm^3^ to 0.489 g/cm^3^) [74]. Both studies also found the concentration of mesopores to increase with bulk density (Thubsuang et al. however did observe a consistently high relative mesopore volume at all densities), and that less-dense samples were instead highly macroporous [34,74]. However, while the above values were observed for acid-catalyzed benzoxazines, heat-treated benzoxazines exhibited different bulk density effects. These samples were primarily macroporous, even at higher bulk densities, to the point where at any density value, micropores did not comprise more than 10% of total pore volume [35]. 

**Figure 22:**
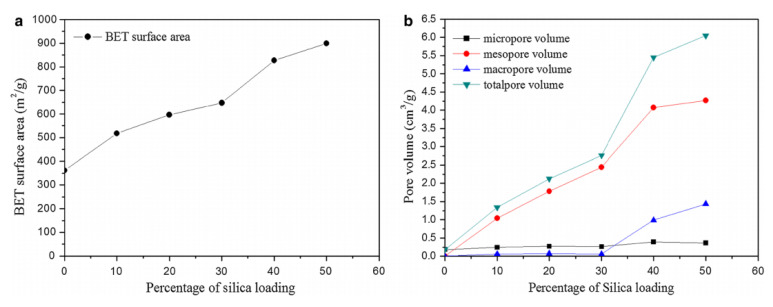
Effects of silica loading on BET surface area (**a**) and pore volume (**b**) of PBZ carbon xerogels (reproduced from the work in [74] with permission).

The use of silica nanoparticles was also found to be beneficial in controlling meso and micropore characteristics. Thubsuang et al. observed that with silica loading of up to 60%, the BET surface area and volumes of all 3 major pore types (especially mesopores) increased dramatically, as seen in Figure 22 [74]. Kim et al. looked at silica nanoparticles in combination with 3-aminophenol and formaldehyde (3-AF) resin during gel formation and observed that the morphology of the resulting carbon gels was particularly dependent on the ratio of 3-AF to silica, where at lower molar ratios of 3-AF to silica, the pore size distribution broadened and morphology of micro-, meso-, and macropores drastically changed [75]. In some respects, however, the presence of silica nanoparticles did not have any significant effect on certain pore characteristics. Thubsuang et al. found that despite the effects on BET surface area and volume mentioned above, the average pore diameter stayed at 24 nm regardless of the amount of silica nanoparticles uptake [74]. This demonstrates that it is generally possible to control porosity using silica templates, especially if higher pore dimensions are desired. 

**Figure 23:**
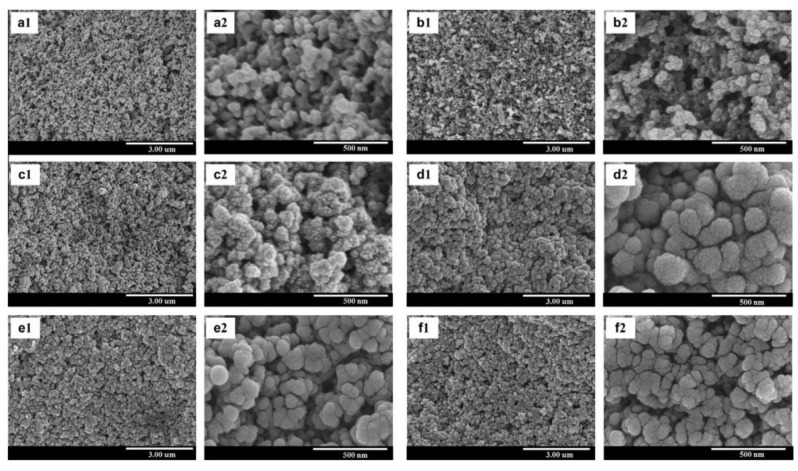
SEM images with low magnification (**1**) or high magnification (**2**) of CTAB added at concentrations of (**a**) 0 M, (**b**) 0.003 M, (**c**) 0.009 M, (**d**) 0.030 M, (**e**) 0.090 M, and (**f**) 0.180 M (reproduced from the work in [36] with permission).

**Figure 24:**
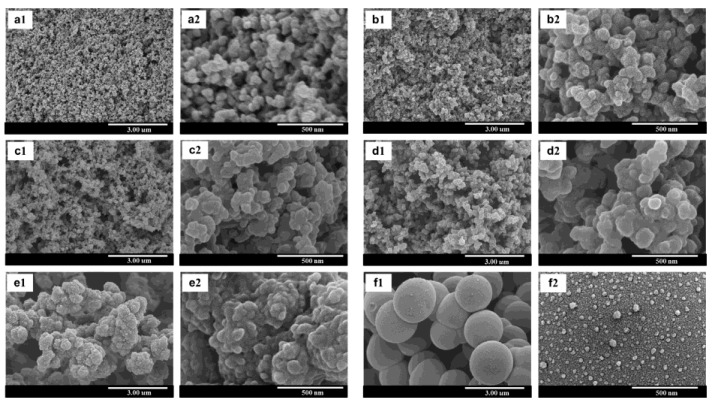
SEM images with low magnification (**1**) or high magnification (**2**) of Synperonic NP30 added at concentrations of (**a**) 0 M, (**b**) 0.003 M, (**c**) 0.009 M, (**d**) 0.030 M, (**e**) 0.090 M, and (**f**) and 0.180 M (reproduced from the work in [36] with permission).

Surfactants have also been found to affect pore distributions, where cationic surfactants tend to yield highly micro- and mesoporous benzoxazines, as opposed to nonionic surfactants which do not impact mesopores as much. Thubsuang et al. used hexadecyltrimethylammonium bromide (CTAB) as a cationic surfactant, which, through its stabilization, led to an even dispersion of silica particles, and in turn yielded higher micropore and mesopore dimensions [74]. A second study by Thubsuang et al. examined nonionic surfactants (polyethylene glycol nonylphenyl ether, shortened to Synperonic NP30) in coordination with CTAB. They found that higher concentrations of CTAB yielded highly mesoporous characteristics, while more Synperonic NP30 resulted in samples with higher amounts of micropores [36]. These morphologies, with either CTAB and Synperonic NP30 added at various concentrations ranging from 0 M to 0.180 M, can be observed in Figure 23 and Figure 24, respectively [36].

The effects of several processes on porosity were also studied including curing, gelation, and drying. Mahadik-Khanolkar et al. found significant shrinkage, as well as an increase in BET surface area, upon curing benzoxazines [35,76]. Crosslinking also dramatically increased BET and micropore surface area, as seen in a study by Abuzeid et al. [73]. The gelation process led to the formation of an interconnected microporous network based on pseudo-gelation point samples taken by Thubsuang et al. [34]. Thubsuang et al. also observed the effects of supercritical drying of carbon xerogels and found that pore characteristics, including BET and micropore surface area and mesopore volume, increased upon supercritical drying [34].

#### 4.2.2. Porous Carbon Nanofibers

Polybenzoxazines can be formed into porous carbon aerogels that are fibrous (as opposed to a solid block), which is a special property of polybenzoxazines. The porosity of aerogels containing CNFs can be easily tailored and also contributes to many aerogel properties. One tailoring method is through CNF activation through a KOH solution, which has the ability to increase porosity of CNFs [37,60]. Si et al. looked at polyacrylonitrile (PAN)-derived bisphenol-A benzoxazine carbon nanofibers embedded with Fe_3_O_4_ nanocrystals (Fe@CNFs) and found that KOH activation led to a significant increase in micropore dimensions (surface area increased by 1256 m^2^/g and volume by 1.404 cm^3^/g), along with a decrease in the ability to graphitize (attributed to the higher number of pores) [37]. Ren et al. found a similar effect on the FeCNF micropore structure by activation, especially through an increase in micro- and mesopore volume (from an average of 0.0337 cm^3^/g to 0.259 cm^3^/g for micropores and 0.654 cm^3^/g to 1.053 cm^3^/g for mesopores), yet the volume fraction of mesopores decreased from approximately 90–100% to 75–86% [60]. This indicates a changed morphology upon activation to increase the amount of micropore and mesopore content in CNFs. 

**Figure 25:**
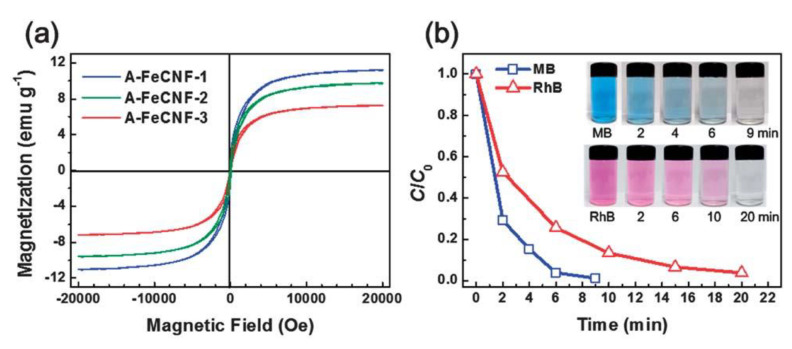
Magnetic hysteresis loops of A-FeCNFs (**a**), dye adsorption over time of A-FeCNFs plotted as the ratio of dye concentration to initial concentration (**b**), and magnetic performance after MB dye adsorption (reproduced from the work in [61] with permission).

The porosity of CNFs can also contribute to other physicochemical, mechanical, and electrochemical properties. The magnetic adsorption capability of FeCNFs has also been studied by Si et al., who found that smaller Fe_3_O_4_ nanocrystals led to increased magnetic adsorption [37]. As both activation and high porosity allow for the Fe_3_O_4_ nanocrystals to grow, the activated Fe@CNFs or more porous FeCNFs were observed to have less paramagnetism (and lower saturation magnetization) [37,60]. Due to their superparamagnetic properties (when prepared at the ideal conditions described above), FeCNFs are suitable candidates for organic dye adsorption from wastewater supplies [37,60]. Figure 25 shows the course of removal of methylene blue (MB) and rhodamine B (RhB) dye particles from water over time, demonstrating the ability of A-FeCNFs to remove all of the MB and RhB dyes in 9 and 20 min, respectively [60].

**Figure 26:**
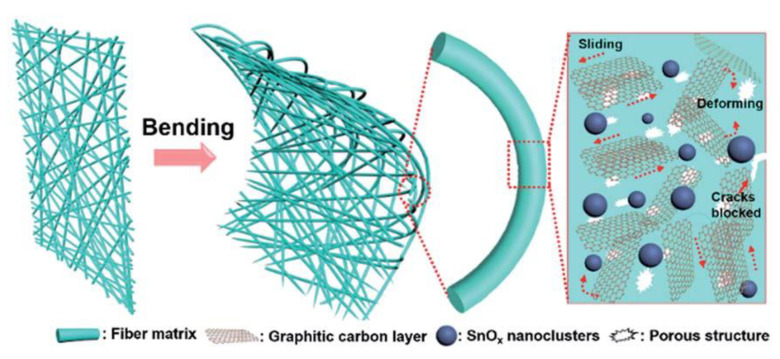
Schematic representation of flexibility of SnO_2_ incorporated CNF membranes under bending (reproduced from the work in [78] with permission).

**Figure 27:**
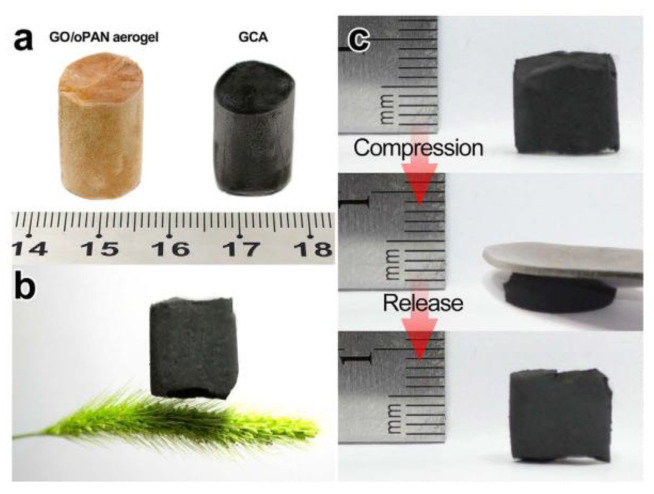
Photographs demonstrating (**a**) shape maintenance, (**b**) low density, and (**c**) and shape recovery capability of GCA (reproduced from the work in [63] with permission).

The mechanical properties of CNF membranes and aerogels have also been studied. Ge et al. found that such membranes were highly elastic and durable, with shape memory properties as well as high stress dissipation [77]. Stress dissipation can be enhanced by uniform distribution of metallic oxide nanoparticles (such as SnO_2_) into the CNF membranes, as this creates a smoother interface between the fibers and the matrix phase [77]. As a result, these membranes had increased the tensile strength, as shown in Figure 26 [77]. Huang et al. also observed high elastic properties in GCAs containing graphene CNFs, which could withstand compressive strains of 70% and recover all strains below 60%, demonstrating superior mechanical properties of GCAs compared to traditional carbon aerogels, which can be observed in Figure 27 [63].

Porous CNFs can also be applied as electrodes, so their current and capacitive properties were examined. The high porosity of the CNFs also allows for better ion transport, thus resulting in materials with high current density (with higher scan rates) that can be observed from CV tests, while GCD tests further demonstrated electrical stability, where 99% of initial capacitance was retained over 10,000 cycles [78]. GCAs were observed to have a slightly lower retention, of 94.8% after 2000 cycles, though this is still higher than other mesoporous carbons [63]. GCAs’ other capacitive properties were improved from that of CNFs, including their charge/discharge time and specific capacitance (180 F/g compared to 76 F/g), indicating that both CNFs and GCAs would be suitable as electrodes [63].

#### 4.2.3. As Electrodes

##### Supercapacitors

As the Anthropocene advances, human dependence on fossil fuels and non-renewable energy sources is an increasingly serious problem. Renewable energy sources, such as wind or solar, are emerging as the future of clean energy generation and will significantly contribute to the world’s energy supply. With global initiatives to reduce emissions, it is critical to advance energy storage and energy conversion technologies such as lithium-ion batteries and supercapacitors [77]. Supercapacitors exhibit high power density, fast charge–discharge processes, and long cycle life, therefore they have applications in mobile electronic devices, electric vehicles, and other renewable energy storage solutions [79,80]. 

**Figure 28:**
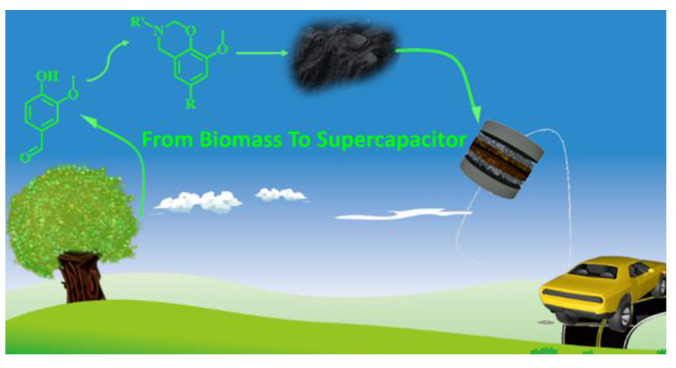
From biomass to supercapacitor (reproduced from the work in [77] with permission).

Microporous carbons, or activated carbons, are commonly used electrode materials for electric double-layer capacitors (EDLCs) as shown in Figure 28. The incorporation of electrochemically active species, such as nitrogen, boron, phosphorus, or oxygen, into the carbon framework can enhance supercapacitor qualities [77,79,80,81,82,83,84,85,86,87,88,89,90,91,92,93,94,95,96]. Introducing heteroatoms into a carbon network can generate reversible pseudo-capacitance from Faradaic electrochemical reactions, which enhances electronic conductivity and surface wettability of the electrolyte and electrode interface and, in turn, improves electrochemical performance of the carbon material [77,80,81,82,84,85,86,87,93,95]. 

Specifically, nitrogen adds to pseudo-capacitance by participating in Faradaic reactions at the electrolyte/electrode interfaces, and improves electrical conductivity, surface wettability, and polarity at the carbon surface [81,95]. Intentionally adding impurities, such as nitrogen, into an intrinsic semiconductor for the purpose of enhancing its electrical, optical and/or structural properties is defined as doping. Doping can be performed in one of two ways: surface modification or in situ doping [87]. Compared to surface modification, in situ doping more evenly incorporates nitrogen atoms into the internal carbon matrix [87]. Benzoxazine intrinsically contains both N and O in the backbone, so utilizing this material is an effective way to exploit both nitrogen and oxygen’s supercapacitor enhancing functionalities, which will be explained in more detail below. Due to the versatile molecular design flexibility of benzoxazine chemistry, doping with other heteroatoms such as S and P, can be readily achieved.

Nitrogen-containing porous carbons (NPCs) derived from nitrogen-containing polymers or biomass derivatives exemplify capacitive behaviors compared to carbon-based materials without pseudo-active species. NPCs exhibit high specific gravimetric capacitance and improved cyclic stability compared to carbon-based materials without impurities. In addition, aerogels feature connected pores as opposed to isolated voids in foams. This enables the use of all the surfaces of pores inside the bulk portion of the material. On another note, NPCs have important applications for acidic gas capture, as they can function as solid adsorbents for CO_2_ or SO_2_. 

However, irregular size and/or dispersion of micropores can slow or prevent ion transport, which causes power density to suffer. Pore size must be optimized in terms of surface area for high energy density but also rapid ion transportation for high power density [79].

Polybenzoxazines are a fitting choice for high-performance nitrogen-doped porous carbons because of their molecular design flexibility, high char yield, good thermal stability, and low shrinkage upon polymerization [83]. Another advantage is that polybenzoxazine aerogels can be manufactured either by freeze drying or ambient drying rather than an expensive supercritical drying process which is unfavorable for scale-up. 

There are two classifications of supercapacitors based on their energy storage mechanism: electric double-layer capacitors (EDLCs) and Faradic pseudo-capacitors [79]. The storage mechanism of EDLCs involves a double layer physisorption of solvated electrolyte ions at the electrolyte/electrode interface. This indicates that the electrode material and its textural/physical properties, including surface area, pore size, electronic conductivity, and surface wettability significantly affect the performance of the EDLCs [80]. Carbon aerogels are applicable supercapacitor electrode materials because of their electrical conductivity, high porosity, controllable pore structure, and highly usable surface area properties [89,90]. Furthermore, when a porous carbon is used as the supercapacitor electrode material, nitrogen doping can improve electrode wettability and electrical conductivity while providing additional pseudo-capacitance [96].

##### Synthesis Techniques

Conventional methods to synthesize nitrogen-doped porous carbons include hard-template, non-aqueous solvent evaporation induced self-assembly, or post-synthesis modification. These methods allow easy manipulation of the mesoporous structure, but involve multiple time-consuming processing steps, have limited batch size and include a costly supercritical drying process [89,96]. Novel synthesis techniques were reported by Katanyoota and Chaisuwan [89], Liu and coworkers [96], and Wan et al. [81] to overcome these shortcomings. Synthesis techniques include ambient drying processes [89], the one-pot aqueous approach [96], and in situ ring-opening polymerization of PBZ in the presence of graphene oxide (GO) [81].

Keeping in mind economic feasibility, Katanyoota and Chaisuwan employed an ambient drying process on two different polybenzoxazine precursors to produce carbon aerogels. The first, BA-teta, was synthesized from bisphenol-A, formaldehyde, and triethylenetetramine (TETA) via a quasi-solventless method, modifying the method reported by Ishida et al. [97]. The second, BA-a, was synthesized from Bisphenol-A, aniline, and paraformaldehyde via the solventless method. Both benzoxazine monomers were dissolved in xylene, poured into vials, then gradually heated to 130 °C for 96 h in an oven. The partially polymerized benzoxazine hydrogels were then dried at room temperature for 2 days, yielding porous organic aerogels. 

**Figure 29:**
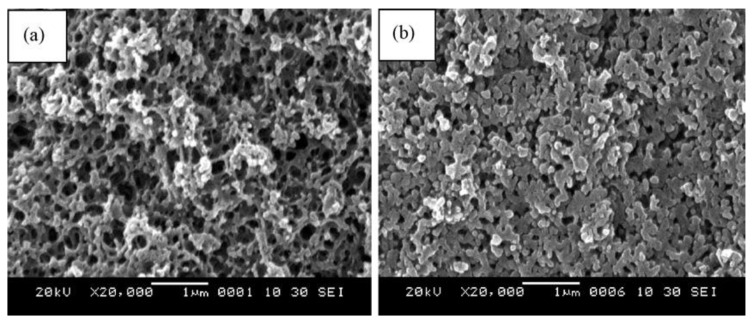
SEM electron photomicrographs of organic aerogel (**a**) and the carbon aerogel derived from it (**b**) (reproduced from the work in [89] with permission).

The thermal properties of the polybenzoxazine precursors reveal that BA-a is more thermally stable because of its aromatic groups, while BA-teta has a higher char yield potentially due to its higher crosslink density. SEM images (Figure 29) reveal that the carbon aerogel (b) has a more dense, three-dimensional porous structure with continuous open macropores compared to the organic aerogel (a) [89].

**Figure 30:**
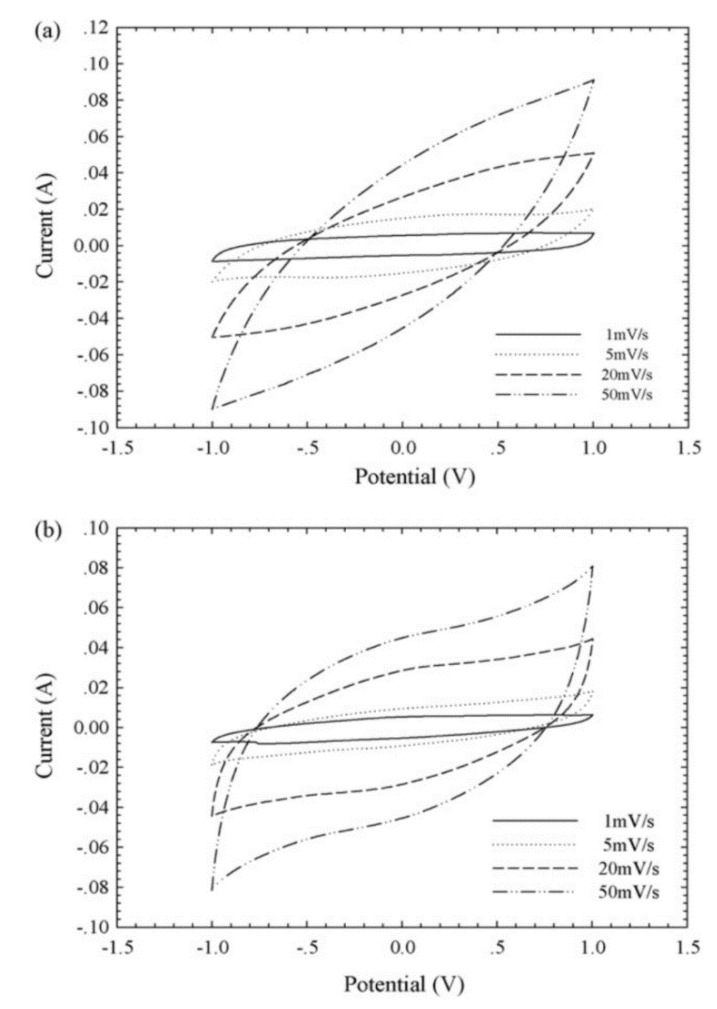
Cyclic voltammograms of CA(BA-teta) (**a**) and CA(BA-a) (**b**) with scan rates of 1, 5, 25, and 50 mV/s (reproduced from the work in [89] with permission).

Cyclic voltammograms (CV) deviate from the rectangular shape at higher scan rates as higher scan rates lessen the amount of time ions must be transported into the pores, shown in Figure 30.

This study successfully used organic polybenzoxazine precursors to prepare carbon aerogels with pore sizes acceptable for electrodes in electrochemical applications. CA(BA-teta) showed approximately twice the mesoporosity and subsequently showed better specific capacitance [89].

**Figure 31:**
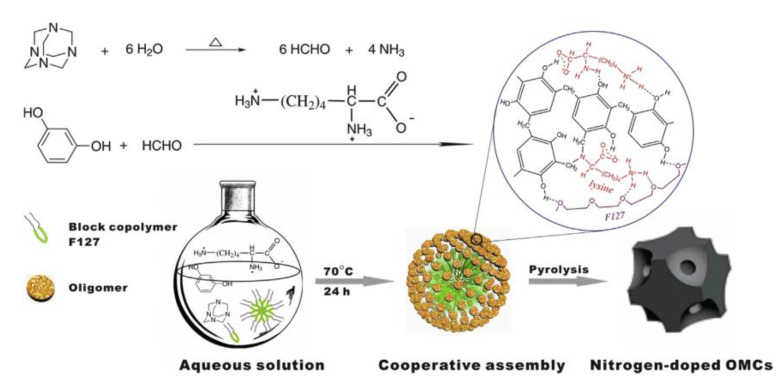
Schematic illustration of L-lysine-assisted formation of cubic nitrogen-doped ordered mesoporous carbons (OMCs) (reproduced from the work in [96] with permission).

Liu and coworkers introduced a highly efficient, one-pot aqueous approach for synthesis of ordered nitrogen-doped mesoporous carbons with tunable composition, as shown in Figure 31.

Specific capacitance (C) can be calculated from the discharge curve by the following equation: *C=itmV*, where *i* is the discharge current (A), *t* is the discharge time (s), *m* is the mass of the carbon aerogel electrode (g), and *V* is the potential difference during the discharge. Functioning as a supercapacitor electrode material with an areal mass loading of ~3 mg cm^−2^, the activated ordered mesoporous carbon (OMC) yields a high specific capacitance of 186 F/g at a current density of 0.25 A/g and good capacitance retention at a high discharge current density of 20 A/g in ionic liquid electrolyte. The authors attribute the electrochemical performance to the synergistic effects of a large surface area, hierarchically ordered micro/mesoporous structure, and nitrogen doping [96]. 

**Figure 32:**
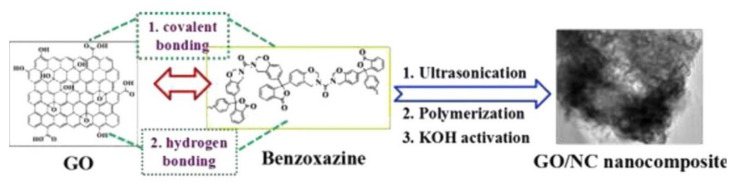
GO/NC nanocomposites prepared from a novel polybenzoxazine (reproduced from the work in [81] with permission).

In regard to the in situ ring-opening polymerization of PBZ in the presence of graphene oxide (GO), Wan et al. synthesized a novel GO and nitrogen-containing porous carbon. The benzoxazine was synthesized from phenolphthalein, urea, and formaldehyde via the solution method. GO and nitrogen-containing (NC) porous carbon nanocomposites, abbreviated GO/NC nanocomposites, were prepared as shown in Figure 32.

A sample achieved a specific capacitance of 406 F/g at 1 A/g, a rate capability of 268 F/g at 40 A/g, and good cycle durability in 6M KOH aqueous electrolyte. These results demonstrate that GO and nitrogen rich PBZ nanoporous carbon composite materials are a promising supercapacitor electrode material. The authors concluded that the amount of GO significantly affects textural properties, composition and surface chemistry, as well as electrical conductivity [81]. Huo et al. [91] also introduced GO into the polymer matrix. Huo et al. investigated GO as a low loading nanofiller for the purpose of producing hybrid materials with improved properties. 

##### Unique Structure

**Figure 33:**
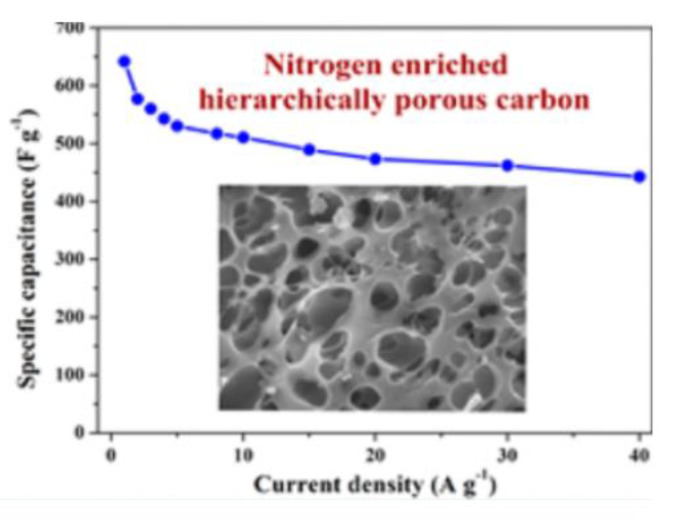
Polybenzoxazine based hierarchically porous carbons (reproduced from the work in [83] with permission).

Polybenzoxazine has a highly tailorable structure which can lead to uniquely structured molecules. Unique structures were reported by Huo et al. [91], Wang and Zhang [92], Wan and Wang [95], Wang and Dong [82], Liu et al. [79], and Wen and Chen [87]. A representative example is shown in Figure 33. Hierarchical pore structures were investigated [82,95] and other porous carbons included GO [91] or surfactant F127 [95]. Hierarchically porous carbons with a distribution of micro-, meso-, and/or macropores reduce in-pore ion transport resistance and diffusion distance, enhancing electrochemical capacitance [83]. 

An important finding among articles is the relationship of synthetic conditions, such as temperature and post treatment, to material properties. What follows are key findings from authors who utilized the high tailorability of polybenzoxazine to create unique molecular structures with good supercapacitor properties. A key finding is that pore structure and surface functionality can be tailored by changing the activation temperature [79,83,93,95] or by using a soft-templating agent such as surfactant, specifically F127 [90,93,95]. Surfactant F127 is a hydrophilic non-ionic surfactant which has applications in cosmetics, bioprinting, and hydrogels.

**Figure 34:**
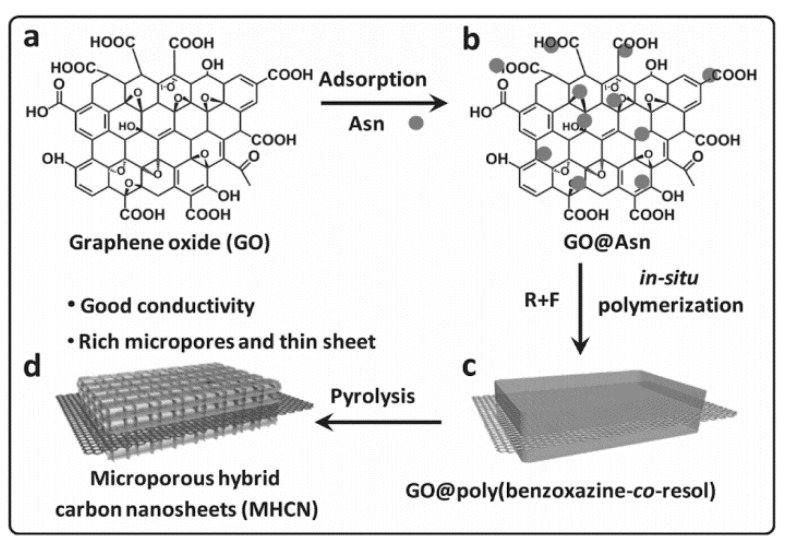
Synthesis principle of MHCN (**a**) negatively charged GO colloids; (**b**) the selected amino acid, asparagine (Asn) dispersed on GO platforms; (**c**) in situ reaction and condensation of pre-loading Asn, resorcinol and formaldehyde, forming poly(benzoxazine-co-resol) layer; and (**d**) formation of microporous hybrid carbon nanosheets during pyrolysis (reproduced from the work in [91] with permission).

The first example of a uniquely structured polybenzoxazine for supercapacitor applications comes from Huo et al. [91]. The group synthesized sandwich-type microporous hybrid carbon nanosheets (MHCN) to enhance supercapacitor performance. A one-atom-thick graphene sheet can be applied as an electrode material for supercapacitors because of its electron mobility and ultra-thin thickness. The group presents a surfactant-free rapid synthesis of MHCN with abundant accessible micropores and thorough graphene percolating by using GO as a shape-directing agent with 2.0–4.0% by weight content as shown in Figure 34.

A sandwiched structure MHCN with high surface area (1293 m^2^/g) and abundant micropores centered around 0.8 nm was effectively synthesized. Rapid diffusion of organic electrolyte ions and the transportation of electrons are made possible by the unique ternary structure. This hybrid ternary structure can elevate energy storage performance across many applications, including supercapacitors [91].

**Figure 35:**
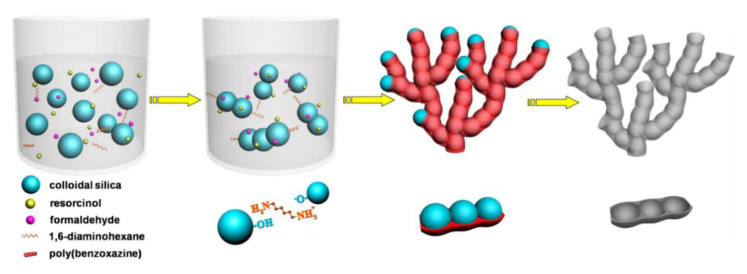
Diagram for the formation process of coral-like carbon (reproduced from the work in [92] with permission).

A research group out of Lu prepared coral-like poly(benzoxazine) porous carbons in the presence of 1,6-diaminohexane for electrochemical energy storage [92]. Formaldehyde was added to resorcinol which was dissolved in deionized water to form a clear solution. Then, a varying amount of a commercially available colloidal silica was added to the solution and heated to 80 °C and vigorously stirred for 42 h. Then, the silica/polymer mixture was pyrolyzed at 800 °C for 2 h under a N_2_ atmosphere. The silica template was dissolved off with aqueous NaOH, leaving a coral-like morphology with a branch-like continuous skeleton containing spherical pore structures as shown in Figure 35.

Wang et al. concludes that when used as a supercapacitor electrode, the carbons exhibit excellent long-term cycle stability, good rate capability with capacitance retention of 88% in the current range of 0.1–0.5 A/g, and almost no capacitance fading after 20,000 cycles at current density of 1 A/g. The carbon materials were also used as a matrix for the encapsulation of SnO_2_ nanoparticles in a lithium storage application. The Li-ion batteries showed high specific capacity and a good cycling stability [92]. 

**Figure 36:**
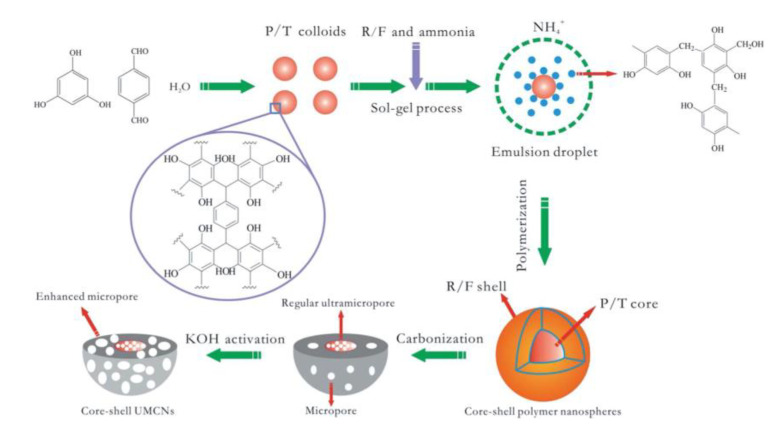
Schematic synthesis of 3D core–shell UMCNs with regular ultra-micropores in the cores and abundant micropores in the shells (reproduced from the work in [79] with permission).

Taking into account the high surface area of combined carbon materials, regular ultra-micropores, and spherical geometry simultaneously, Liu et al. [79] expects an innovative material tailored for high-performance EDLCs. Liu et al. designed and synthesized ultramicroporous@microporous carbon nanospheres (UMCN) with a unique 3D core-shell with the potential to be used as advanced supercapacitor electrodes. By time-controlled polymerization of phloroglucinol and terephthalaldehyde (P/T), polymer colloids were prepared, and then resorcinol/formaldehyde (R/F) copolymerized on the colloid surfaces to fabricate nanospheres. Carbonization and further KOH activation generates regular ultra-micropores in the inner (P/T) cores and abundant micropores in the outer (R/F) shell as depicted in Figure 36. 

**Figure 37:**
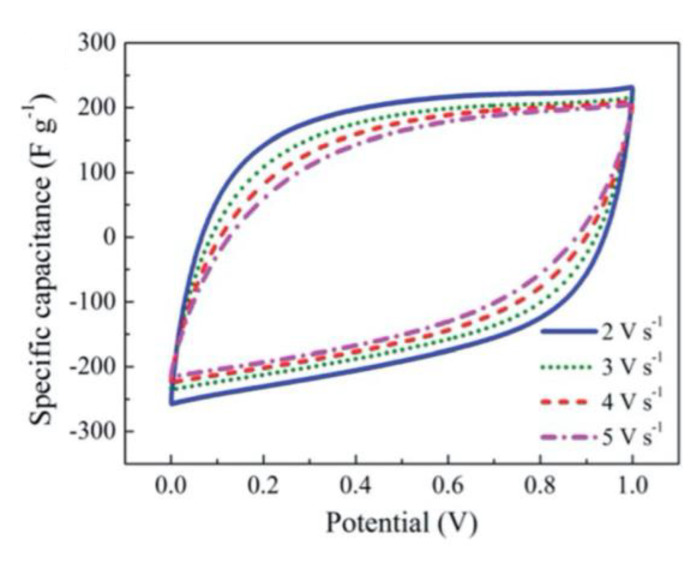
CV scan of UMCN-60 electrode at scan rate of 5 V/s (reproduced from the work in [79] with permission).

The rectangular shaped cyclic voltammogram (CV) of electrode sample UMCN-60 displays fast ion-transport and good capacitive performance for quick charge–discharge operation as seen in Figure 37. 

Resulting unique nanoarchitecture UMCNs exhibit high gravimetric capacitance (411 F/g at current density of 1 A/g), ultra-high rate capability, excellent long term cycle life and high power density (50 kW/kg at 100 A/g) [79].

A uniquely structured asymmetric supercapacitor with excellent performance and low cost was fabricated by Wen and Chen [87]. Asymmetric supercapacitors consist of a negative potential electrode and another electrode that stores energy at a positive potential. Advantages include higher energy and power density due to the larger potential window [87]. Asymmetric supercapacitors have potential applications in renewable-energy technology because of their electrochemical properties. Porous carbons, metal oxides, and conducting polymers are the most commonly used electrode materials for supercapacitors [86]; of metal oxides, ruthenium oxide, cobalt oxide, nickel nitrate, manganese dioxide (MnO_2_), and vanadium oxide have been used. Among them, MnO_2_ has the most advantages, as it is non-polluting, has high theoretical specific capacitance, is abundant, and has easily controllable morphology and structure. Wen and Chen [87] prepared MnO_2_ petal nanosheets grown on carbon sphere surfaces as a cathode material. The anode material was a nitrogen-doped activated carbon prepared from a bisphenol-A type benzoxazine. 

Wan et al. synthesized an NPC with hierarchical pore structure from a benzoxazine monomer precursor through a soft-templating method and KOH chemical activation. The precursor was a novel bifunctional benzoxazine with nitrile functionality, synthesized from 4-cyanophenol, melamine, and formaldehyde by the Mannich condensation reaction via a solution method.

As previously mentioned, a soft templating agent, such as surfactant, F127, or activation temperature, can easily tailor NPC pore structure. To investigate the efficacy of these polybenzoxazine based NPCs, [95] tested electrochemical behavior in 6 M KOH aqueous electrolyte and CO_2_ capture performance was tested at ambient pressure. At 1 bar, sample NPC-1 achieved CO_2_ uptakes of 6.20 and 3.95 mmol/g at 0 and 25 °C, respectively, as tabulated in Table 1.

NPC-1 and NPC-2 exhibited improved capacitive performance due to their high surface area, appropriate pore size distribution, good electrical conductivity, and presence of N and O functional groups when compared to sample NPC-0. High-rate capability, electrochemical stability, and high specific gravimetric capacitance (362 F/g at 1 A/g) were exhibited by sample NPC-2. These results allowed the authors to conclude that such nitrogen-containing porous carbons are promising supercapacitor electrodes and superior adsorbents for the capture of CO_2_ [95].

**Figure 38:**
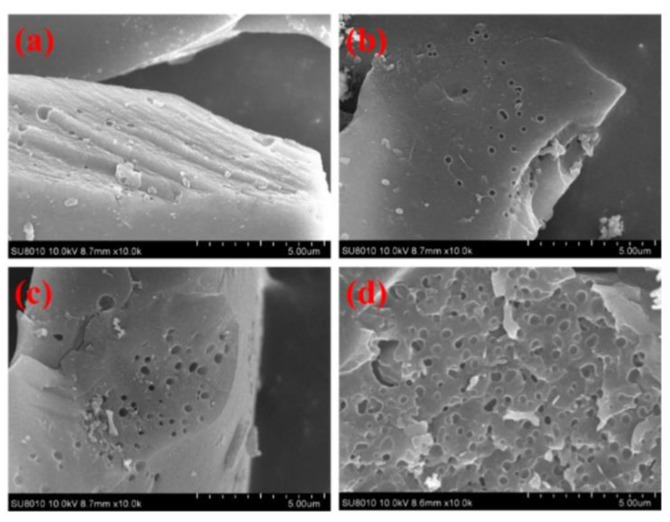
SEM images of (**a**) NPC-500, (**b**) NPC-600, (**c**) NPC-700, and (**d**) NPC-800 (reproduced from the work in [83] with permission).

Wang and coworkers [83] prepared polybenzoxazine from phenol, aniline, and paraformaldehyde via a solventless method and thermal ring-opening polymerization. To demonstrate the effect of KOH activation temperature on structural properties, scanning electron microscope (SEM) imaging was performed. Changing the KOH activation temperature from 600 °C to 800 °C increased the number of pores formed and their diameter as depicted in Figure 38. 

The HPC-800 sample activated at 800 °C achieved the highest specific capacitance of 402 F/g at 0.1 A/g and 249 F/g at 10 A/g, and more than a 99.0% capacitance retention ratio after 500 charge/discharge cycles in a three-electrode system [83].

##### Multi-Element Doping

Co-doped carbon electrodes were investigated by Liu and Cao [77] (N&O), Zhang et al. [84] (N&P), Yan et al. [85] (N&P), and Bai et al. [86] (B&N). Strictly, doping is defined as the intentional addition of impurities into the bulk. The addition of impurities into the bulk can be interpreted as the existence of impurities. In this case, the nitrogen and oxygen in benzoxazine are pre-existing impurities, so the term N-doped or N- and O-doped refers to the existence of impurities rather than the addition. In addition to N and O, other elements such as P [62,63] and S have been purposely added by molecular design.

An interesting paper by Liu and Cao presented a nitrogen and oxygen co-doped material prepared from biobased polybenzoxazine by a soft-templating method. The authors synthesized two different biobased benzoxazines from renewable vanillin, one containing cyano groups and the other without. The cyano group adds extra nitrogen atoms, which as previously discussed, will influence pore structure and conductivity. This work takes a large stride towards sustainability as the authors designed renewable materials with promising electrochemical performance for energy applications [77].

For the goal of increasing packing density and specific capacitance of porous carbon materials, nitrogen, and phosphorus co-doping is investigated by many groups. Zhang et al. investigated a nonporous polybenzoxazine co-doped with nitrogen and phosphorus. Phosphorus doping occurs at the edge of the graphitic framework while nitrogen doping occurs at the basal and edge plane sites [84]. It is the combination of nitrogen and phosphorus at the edge plane that enhances specific capacitance by means of pseudocapacitance. Melamine polyphosphate was used as a nitrogen and phosphorus co-doped precursor and the polybenzoxazine was prepared by polymerization of 1,3-benzoxazine and then used as an in situ nitrogen-doped carbon precursor. One sample achieved a specific capacitance of 203 F/g at 0.5 A/g and high capacitance retention of 90.1% after 5000 cycles at 5 A/g [84]. 

**Figure 39:**
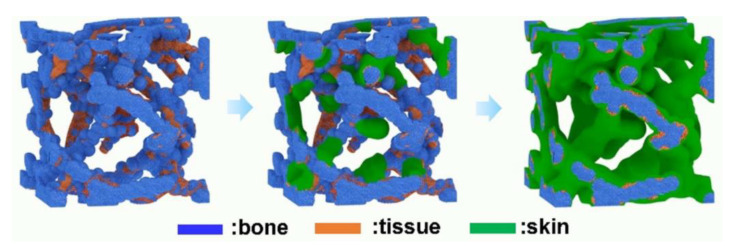
Schematic representation of the skin-tissue-bone like structure (reproduced from the work in [85] with permission).

Yan et al. utilized ionic liquid, C_16_mimPF_6_, for the self-assembly of polybenzoxazines and obtains a unique skin-tissue-bone-like hierarchical porous carbon with homogeneous N and P co-doping as shown in Figure 39. 

Both hierarchical pore structure and the introduction of heteroatoms are effective ways to improve supercapacitor performance. However, such materials are challenging to obtain by a one-step synthesis procedure. C_16_mimPF_6_ works as both a structure-directing agent and a heteroatom precursor. KOH activation is unfavorable for scaling up because it is corrosive, expensive, and not eco-friendly. The dramatic interaction between C_16_mimPF_6_ and surfactant F127 creates a microphase separation mechanism. Hierarchical nanostructure is coined skin-tissue-bone structure by the authors [85].

Small energy densities, in the range of 5 to 10 Wh/kg, restrict traditional porous carbons for practical application in energy capture and storage. In recent years, many efforts have been made to incorporate electron-deficient boron and electron-rich nitrogen into carbon materials for supercapacitors. For example, Bai et al. prepared boron and nitrogen co-doped porous carbons (BNCP-X) from boron-containing polybenzoxazines by carbonization and KOH chemical activation. Results show that the BNCP-0.15 possess 2.97 wt % boron and 2.43 wt % nitrogen, a homogeneous pore distribution, high specific capacitance (286 F/g at 0.05 A/g), good rate capability, and a charge–discharge stability greater than 92% capacitance retention after 1000 cycles at 1.0 A/g in 6M KOH solution [86]. 

##### Use of Soft Templating Agent

The use of surfactant F127 as a soft-templating agent was reported by Guo et al. [90], Wan and Wang [93], Wan et al. [95], and Yan et al. [85]. Generally, post-modification processes are simple but entropic in terms of both the amount and distribution of dopants. Ionic liquids (ILs) have been utilized as carbon precursors via cyclotrimerization to form a triazine network, and then converted to N-doped porous carbon materials under pyrolysis. Guo et al. [90] attempts to employ ionic liquids as a heteroatom doping agent source during the polymerization of carbon precursors. The ionic liquids must contain crosslinkable functional groups. C_16_mimBF_4_ is a B- and N-containing ionic liquid which Guo et al. used for the preparation of poly(benzoxazine-co-resol)-based monolithic carbons. C_16_mimBF_4_ and surfactant, F127, were added to the polybenzoxazine made from resorcinol, formaldehyde, and 1,6-diaminohexanamin via a self-assembly polymerization method to improve the electrochemical performance. In all samples, the molar ratio of resorcinol/surfactant was 275:1 and resorcinol/formaldehyde at 1:2 [90]. 

C_16_mimBF_4_ incorporates boron into the framework and modulates the carbon microstructure. One of the samples showed excellent capacitance performance and long term cyclability with a high C_g_ (247 F/g), C_s_ (66 F/cm^2^), and C_v_ (101 F/cm^3^) at a constant 0.5 A/g current [90].

**Figure 40:**
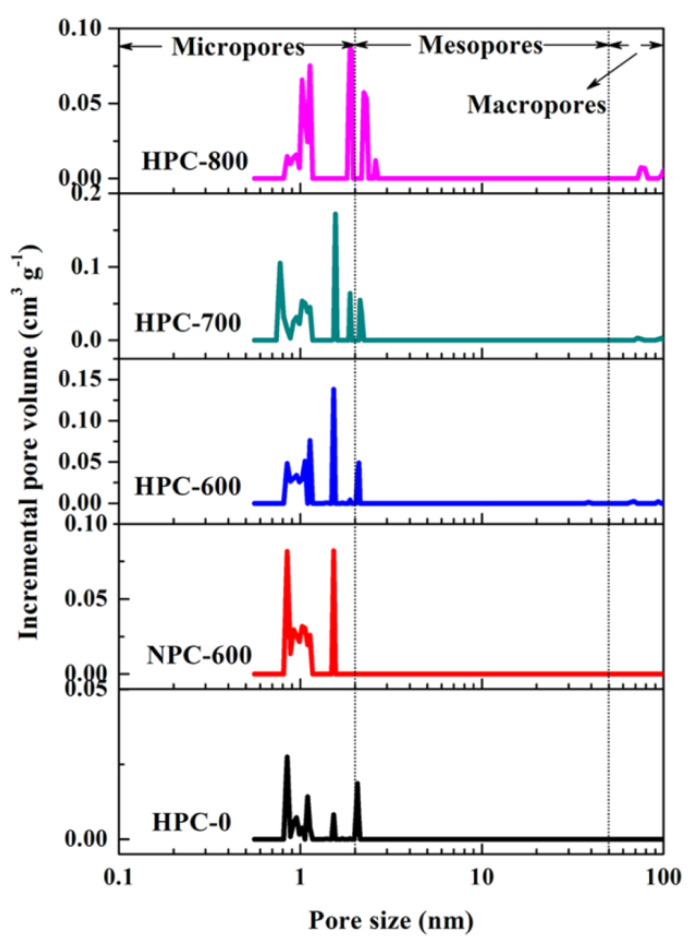
PSDs obtained from DFT method of all samples (reproduced from the work in [93] with permission).

Wan et al. [53] synthesized hierarchically porous carbons (HPCs) from a novel nitrile-functionalized polybenzoxazine for high-performance supercapacitor applications. 4-Cyanophenol, diamine urea, and formaldehyde were used to synthesize nitrile-functionalized benzoxazines via a solution method. The authors note that HPCs with well-defined and interconnected micropores, mesopores, and macropores are promising electrode materials for supercapacitors, specifically those which can exhibit combined advantages of all three pore sizes. Figure 40 depicts PSDs of all three pore sizes obtained by the density-functional theory (DFT) method of all samples studied by Wan et al. In addition, the surfactant and activation temperature were reported to play an important role in resulting textural properties and surface chemistry [93].

Wang et al. [88] investigated ultrasonication time, benzoxazine monomer concentration, and carbonization temperature effects during the preparation process of a meso porous carbon material. The N-doped mesoporous carbons were synthesized from a 1,6-hexanediamine, formaldehyde, and phenol benzoxazine precursor. SBA-15 was used as a template and the CNM-800 material had the highest specific capacitance value at 429 F/g at 0.25 A/g in a three electrode system [88].

##### As Materials for Batteries 

The primary source of greenhouse gas emissions in the United States comes from the transportation sector [98]. The majority of transportation-related emissions are carbon dioxide (CO_2_) emissions from petrol and diesel fuel vehicles [98]. The emergence of electric cars is not just an ecofriendly goal, but rather a business reality to combat climate change. The core component of electric vehicles (EVs) and hybrid electric vehicles (HEVs) is the rechargeable battery [99,100,101]. Compared to other rechargeable batteries like Ni-Cd, Ni-MH, and lead-acid batteries, the lithium-ion battery (LIB) has high energy and power density, long service life, and environmental friendliness [101]. 

Currently, graphite serves as the anode material in commercial LIBs, however the theoretical capacity, solid-state diffusion coefficient, and rate performance are low [99]. Porous carbons allow for many active Li-ion storage sites and have high available capacity and negligible volumetric expansion [99]. Common problems with cathode materials such as lithium iron phosphate (LiFePO_4_) [99] or Li-S [102,103,104,105] are low intrinsic electronic conductivity and slow diffusion of Li-ions across the boundary. Lithium iron phosphate (LiFePO_4_) was studied as a cathode material because of its excellent electrochemical properties [99]. However, the material is limited by low intrinsic electronic conductivity and slow diffusion of Li-ions across the boundary. 

A short Li diffusion path and a high electrode/electrolyte contact surface area are the key factors to improve the capacity of porous carbon anodes [99]. The diffusion formula is given as *t=L^2^/2D* where t is the diffusion time, L is diffusion distance, and D is the diffusion coefficient. This formula shows that reducing particle dimensions can significantly shorten diffusion time, therefore enhancing power performance. Most published work has studied the incorporation of mesopores in order to effectively reduce Li diffusion time. Mesopores allow for rapid transport channels. However, some studies have paid attention to the effect of volume ratio between mesopores and micropores (V_mes_/V_mic_) [99]. A high V_mes_/V_mic_ ratio favors diffusion of Li-ions to active sites.

Due to increasing energy storage demands for portable electronics and electric vehicles, researchers have been working to develop higher energy density storage systems. Li-S batteries have been studied as a potential next-generation energy storage system [102,103,104,105]. When used as the cathode material, S offers a high theoretical capacity of 1675 mAh/g [102], high theoretical energy density, high theoretical specific energy (~2600 Wh/kg), low cost, non-toxicity, and abundance [105]. However, there are many drawbacks that have prevented the practical application of Li-S batteries [102,103,104,105]. High sulfur load cathodes exhibit rapid capacity fading caused by the “shuttle effect”. The “shuttle effect” describes the easy dissolution of polysulfide intermediates between the two electrodes, and it remains a problem even with attempts to overcome it. Pei et al. [102,103,104,105] has employed sulfur hosting materials to increase the utilization of sulfur and alleviate polysulfide loss from cathodes. Porous carbons are particularly apt as the sulfur-hosting material, and polybenzoxazine has been incorporated. For example, Pei et al. [105] reports a functional separator made of 2D porous nitrogen-doped carbon nanosheets. In this application, the authors took advantage of polybenzoxazines high tailorability and synthesized a conductive, lightweight barrier with polysulfide-trapping abilities to suppress shuttling of polysulfides. 

#### 4.2.4. As Adsorbents

##### CO_2_ Capture

**Figure 41:**
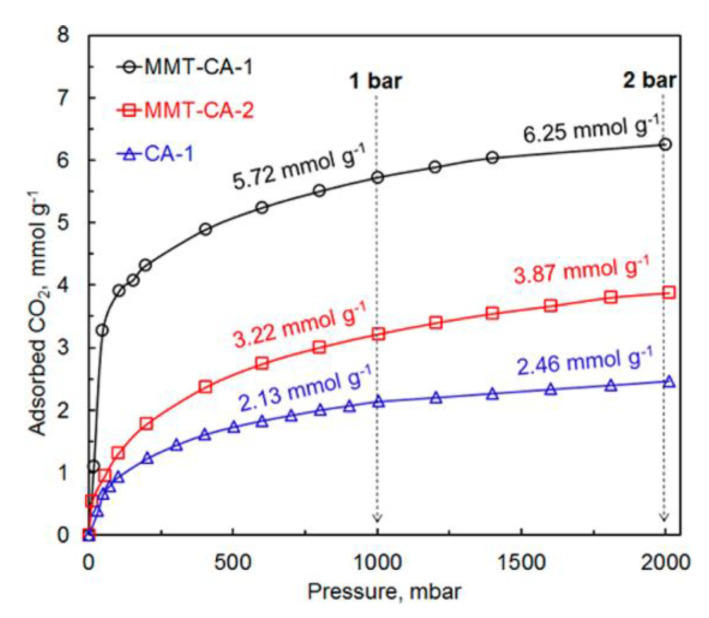
CO_2_ adsorption of MMT-CA-1 (highest micropore volume), MMT-CA-2 (highest BET surface area), and CA-1 benzoxazine samples with varying clay concentrations (reproduced from the work in [37] with permission).

Within the past decade, polybenzoxazines have been found to be extremely effective in adsorbing CO_2_ from air streams, which is crucial for reducing industrial pollution and weakening the effects of global warming from carbon emissions [37,106,107,108,109,110,111]. Many studies attributed this ability to the porous qualities of benzoxazine, particularly the micropore volume and BET surface area. Micropore volume, which originates from the release of degradation products during carbonization [109], is the primary driving factor behind CO_2_ adsorption [37,107]. Higher micropore volumes allow for increased CO_2_ adsorption, as observed by the trend in Figure 41 showing that the highest CO_2_ uptake at 1 bar of 5.72 mmol/g occurred in the benzoxazine with the highest micropore volume of 0.319 cm^3^/g, a montmorillonite carbon aerogel with a main-chain BA-tepa weight ratio of 1 (MMT-CA-1) [37,107,111]. This phenomenon was demonstrated both theoretically and experimentally by Far et al., who found that the experimentally obtained adsorption was close to the theoretical adsorption based on micropore volume for nearly all benzoxazine samples, and that the theoretical and experimental comparison was most accurate for micropore volume calculations rather than BET surface area calculations, indicating a strong positive effect of micropore volume [107]. Wu et al. looked at average pore diameter, which is closely related to pore volume, and found that slight changes in diameter led to higher CO_2_ adsorptions, confirming the strong effect of volume [106]. As mentioned previously, other pore characteristics, namely, BET surface area followed by pore diameter, also play a role in CO_2_ uptake. Wu et al. and Mohamed et al. observed a completely positive trend between BET surface area and CO_2_ adsorption, where dramatic increases of BET surface area (from 1667 m^2^/g to 1866 m^2^/g for Wu et al.’s study) led to a near doubling in adsorption capacity [106,110,111]. However, other studies observed opposite effects, particularly Alhwaige et al., as illustrated by Figure 41, where the benzoxazine sample with higher BET surface area at 710 m^2^/g had significantly lower adsorption capacity than MMT-CA-1, the sample with a surface area of 679 m^2^/g. [37]. While BET surface area may be a factor in CO_2_ uptake, it is not as consistently significant as micropore volume [37,107]. Micropore volume and BET surface area are the dominant characteristics driving CO_2_ adsorption, though there are numerous other factors, including processes and conditions.

**Figure 42:**
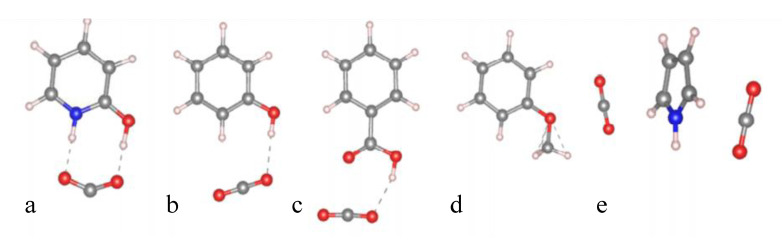
CO_2_ interactions with substrates (in the carbonized benzoxazine) containing (**a**) pyridonic nitrogen, (**b**) hydroxy groups, (**c**) carboxylic acids, (**d**) methoxyl, and (**e**) pyrrolic nitrogen, with the effect of hydrogen bonding shown in a–c (reproduced from the work in [108] with permission).

One of these alternate factors was hypothesized to be hydrogen bonding, as proposed by Hong et al. The presence of hydrogen bonding decreases the required binding energy for CO_2_ on the active sites in the benzoxazine micropores, thus providing an anchoring effect between the CO_2_ and benzoxazine [108]. This was also said to be the reason for CO_2_ selectivity over N_2_, as N_2_ is unable to form as strong intermolecular forces [108]. Multiple schemas of the bonding of CO_2_ can be seen in Figure 42.

As mentioned previously, several processes can influence CO_2_ adsorption, including KOH activation. Wu et al. found that activation increased the concentration of nitrogen in doped and undoped benzoxazine as well as altered the microporous structure, which were both found to correlate with adsorption capacities [106]. Mohamed et al. also saw the increased concentration of nitrogen and oxygen in benzoxazines after KOH activation, which positively correlated with CO_2_ capture [110]. This outcome was also contributed to by the increased porosity (micropore diameter and BET surface area) that resulted from activation [109]. A study by Mohamed et al. also found a higher microporosity upon activation, as well as increased nitrogen content in various forms [111]. Hong et al. looked at the activation temperature in addition to the presence of KOH activation and found that a higher activation temperature led to increased porosity and higher BET surface areas [108]. They observed that experimental CO_2_ uptake increased with activation temperature until the highest activation temperature of 900 °C, where it dipped slightly (by approximately 0.27 mmol/g) from its highest value at an activation temperature of 800 °C, despite micropore volume continually increasing [108]. On the other hand, Konnola et al. found that activation did not necessarily play a role in CO_2_ adsorption capabilities. An unactivated benzoxazine sample was able to capture 3.17 mmol/g of CO_2_, which is higher than ten literature values of CO_2_ capture from activated carbon, the highest reported value of which, in the study by Konnola et al., was 3.1 mmol/g [109]. This demonstrates that it is very possible to use unactivated benzoxazine for CO_2_ adsorption, despite activated carbon being more common. 

Additional processes besides KOH activation were found to influence porosity, and thus CO_2_ uptake capabilities. These processes include curing, carbonization, and etching of the benzoxazine. Alhwaige et al. looked at both the curing and carbonization processes, and found that CO_2_ uptake correlated positively with carbonization, but negatively with curing [37]. The negative effect of polymerization is hypothesized to be due to the reduction of sites for CO_2_ by the formation of crosslinks, despite them increasing mechanical stability [37]. Carbonization, on the other hand, led to an 8-fold increase of CO_2_ adsorption capability when non-carbonized and carbonized benzoxazine samples were compared [37]. Polybenzoxazine samples were etched through pyrolysis, which smoothed out the micropore cavities (despite not changing micropore distribution) and was found to dramatically increase the porosity of each sample [107]. BET surface area in particular became significantly larger after etching (as large as 2300 m^2^/g to 2800 m^2^/g), and as it has been found that BET surface area led to dramatic increases in CO_2_ uptake, the adsorption abilities of the etched benzoxazines are thus theoretically higher than the unetched ones [107]. The experimental results observed by Far et al. confirm this, where for every type of benzoxazine tested, the CO_2_ uptake of the etched version was at least as large, if not significantly larger, than the unetched one [107].

**Figure 43:**
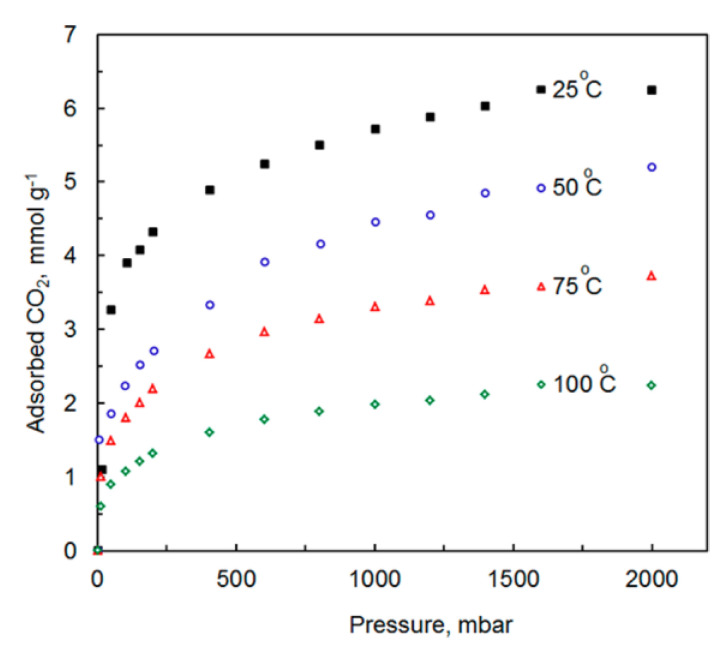
Breakthrough curves of MMT-CA-1 at increasing temperatures (reproduced from the work in [37] with permission).

Temperature and pressure effects on CO_2_ adsorption were also studied. The breakthrough curve in Figure 43 demonstrates that despite pressure being positively correlated with adsorption, increasing temperatures were found to have lower levels of CO_2_ adsorption, particularly the initial capacities at 0 mbar [37]. Alhwaige et al. explain that the decrease of adsorption capacity is due to CO_2_ adsorption being an exothermic process [37]. Hong et al. measured CO_2_ adsorption at 25 °C in addition to 0 °C. It was found that for every activation temperature, the CO_2_ uptake was higher at 0 °C than at 25 °C, indicating that increased temperature has a negative effect on gas uptake [108]. Konnola et al. used similar conditions for testing CO_2_ adsorption and again found that adsorption was higher for all samples tested at 0 °C, with a maximum value of 4.25 mmol/g, rather than at 25 °C, with a maximum value at 3.17 mmol/g [109]. This confirms the conclusion by Alhwaige et al. that CO_2_ adsorption was exothermic [37]. Mohamed et al. also observed this temperature dependence, where nitrogenated benzoxazine samples were able to adsorb over 2 times as much CO_2_ at 0 °C than at 25 °C. An increase in adsorption at 0 °C compared to 25 °C was also observed for un-nitrogenated samples, though the difference, from 3.30 mmol/g to 5.25 mmol/g, was not nearly as dramatic [110]. This was again observed in another study, where both benzoxazine samples studied had higher CO_2_ uptake by approximately 1–2 mmol/g, depending on the sample at 0 °C instead of 25 °C [111].

As described previously, CO_2_ uptake did not always increase with higher porosities, indicating that there may be other factors that contribute to gas adsorption. Hong et al. proposed that hydrogen bonding played a key role in CO_2_ uptake. The presence of hydrogen bonding decreases the required binding energy for CO_2_ on the active sites in the benzoxazine micropores, thus providing an anchoring effect between the CO_2_ and benzoxazine [108]. This was also said to be the reason for CO_2_ selectivity over N_2_, as N_2_ is unable to form as strong intermolecular forces [108].

The presence of heteroatoms, primarily nitrogen [37], further contributes to CO_2_ adsorption, commonly through heteroatom effects on pore dimensions. Wu et al. observed this by comparing the adsorption abilities of nitrogen doped benzoxazines (BZCN) and undoped benzoxazines (BZPh) [106]. Pore qualities of BET surface area and pore diameter both increased in the nitrogen-doped benzoxazine, relative to the undoped as listed previously, which in turn led to increased CO_2_ adsorption [106]. Hong et al. found nitrogen to have a significant effect in the pyrrolic or pyridonic forms, though these forms were not as abundant in the benzoxazine samples, as opposed to pyridinic nitrogen [108]. Nitrogen-containing samples studied by Mohamed et al. were also found to have significantly higher BET surface areas and pore diameters than samples with lower amounts of nitrogen and were thus able to adsorb much more of CO_2_ as the undoped samples [110]. Mohamed et al. observed a very significant effect by the presence of nitrogen, especially in pyridonic and pyridinic forms [111]. They found that when the nitrogen content increased from 2.3 wt% to 4.2 wt%, the CO_2_ uptake increased from 4.60 mmol/g to 7.20 mmol/g (at 0 °C, though a similar increase was observed at 25 °C) [111]. These studies all demonstrate that nitrogen plays a significant role in CO_2_ capture in addition to porosity, indicating some favorability with CO_2_ and nitrogen. 

**Figure 44:**
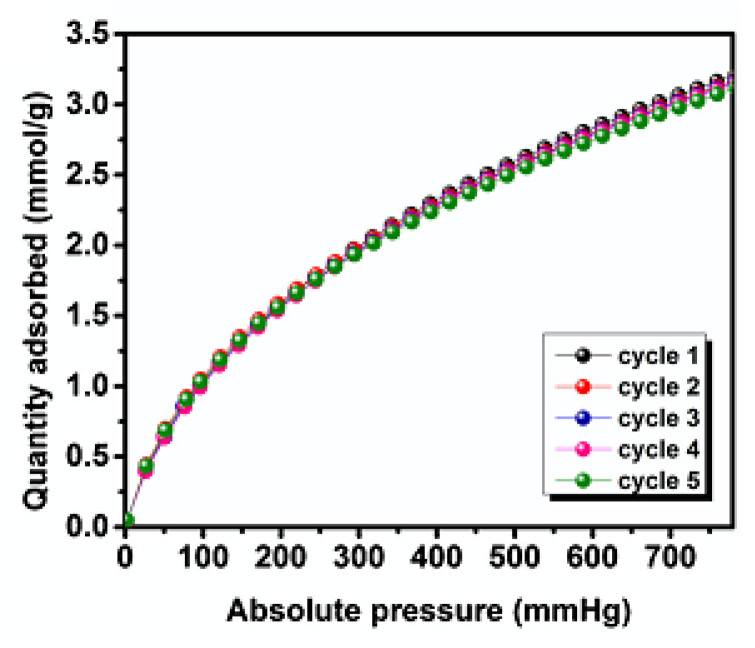
CO_2_ adsorption of N- and S-doped benzoxazines over 5 cycles (reproduced from the work in [109] with permission).

Aside from the large effect of nitrogen on CO_2_ adsorption, other heteroatoms, namely, oxygen and sulfur, also played a role. Far et al. looked at benzoxazines doped with oxygen or nitrogen- and oxygen-doped benzoxazines and found that, unlike previously studied, the samples that were not nitrogen-doped adsorbed more CO_2_, though this may be due to the fact that the amount of nitrogen decreased by half upon etching while the amount of oxygen remained mostly constant [107]. This indicates that nitrogen was likely the more influential heteroatom in CO_2_ adsorption and that etching subsequently had a larger effect than anticipated. Hong et al. also observed higher CO_2_ adsorption in benzoxazines containing more oxygen as increased oxygen content, specifically in the form of hydroxy functional groups, led to polarity of the benzoxazine, which strengthened the hydrogen bonding effects involved in CO_2_ capture [108]. Due to this capability, Hong et al. explained that oxygen had significant effects, aside from those contributed by nitrogen, and that oxygen doping had been previously overlooked [108]. Nitrogen and oxygen doped benzoxazine samples studied by Mohamed et al. had higher CO_2_ uptakes at both temperatures observed, and Mohamed et al. concluded that benzoxazine doped with these two heteroatoms would be useful for CO_2_ capture from air streams [110]. Konnola et al. studied dual-doped nitrogen and sulfur benzoxazines. They observed that the smooth dispersion of sulfur and nitrogen in the benzoxazine network led to highly porous materials that subsequently had high CO_2_ adsorptions, ranging from 3.52 mmol/g to 4.25 mmol/g at 0 °C in various samples [109]. It was also found that isosteric heats of adsorption were higher for nitrogen and sulfur containing samples, indicating that these heteroatoms had an effect on the favorable interactions between the benzoxazine and CO_2_ [109]. Figure 44 further highlights the stability of nitrogen- and sulfur-doped benzoxazines, which maintained their CO_2_ adsorption capacities at 25 °C for up to 5 cycles [109]. 

**Figure 45:**
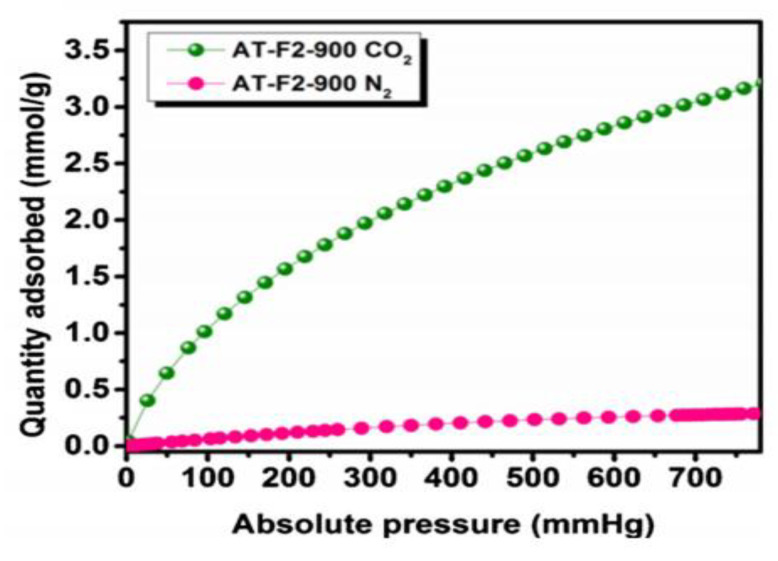
CO_2_ and N_2_ adsorption by benzoxazines at 25 °C (reproduced from the work in [109] with permission).

The uptake capabilities of other gases, especially H_2_, N_2_, and CH_4_, by benzoxazine-derived carbon aerogels derived from polybenzoxazines was also studied, with special attention paid to the ability to separate CO_2_ from these other gases. This selectivity is especially important if polybenzoxazine-based carbon was to be used for a CO_2_-specific air filtration application. Far et al. found that benzoxazine-derived carbon had a high selectivity towards CO_2_ from H_2_ and N_2_, though not CH_4_ [107]. The lack of selectivity with CH_4_ is hypothesized to be due to the polarizability of CH_4_ [107]. On the other hand, Hong et al. did find good selectivity with CO_2_ from CH_4_ based on breakthrough curves that showed CO_2_ adsorption, at 8.44 mmol/g, was nearly four times the amount of CH_4_ adsorption at 2.18 mmol/g [108]. They also observed similar performance for the uptake of N_2_ and CO_2_ for samples at both testing temperatures and all activation temperatures, which can be attributed to the hydrogen bonding present in CO_2_ uptake [108]. This selectivity for CO_2_ from N_2_ was also observed by Konnola et al. who observed that the CO_2_ uptake at 3.17 mmol/g was over 11 times higher than N_2_ uptake, at 0.28 mmol/g, as seen in the breakthrough curve in Figure 45 [109].

Studies used ideal adsorption solution theory (IAST) to predict the selectivity of CO_2_ over other gases. IAST selectivity factors (S) are calculated from the amounts of CO_2_ (*q*_1_) and N_2_ (*q*_2_) adsorbed at their partial pressures (*p*_1_ and *p*_2_, respectively) as

*S* = *q*_1_*p*_2_*q*_2_*p*_1_ (1)

**Figure 46:**
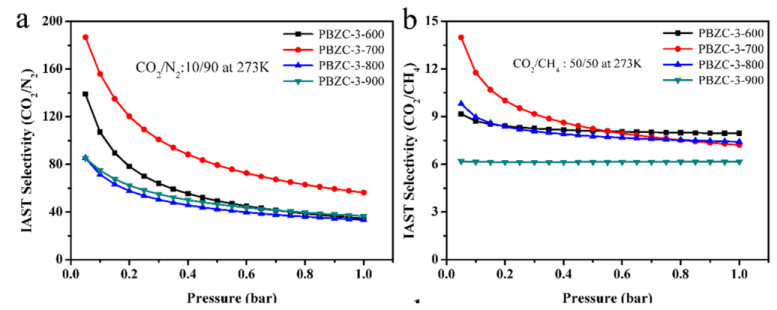
IAST selectivity factors of CO_2_ adsorption from (**a**) N_2_ and (**b**) CH_4_ (reproduced from the work in [108] with permission).

IAST selectivity factors calculated by Hong et al. confirm the high selectivity of CO_2_ from N_2_, where the selectivity factor was above 20 at 0 ℃ for all benzoxazine samples and was particularly high at 60 for benzoxazine activated at 700 °C, as shown in Figure 46 [108]. Methane, however, demonstrated low selectivity from CO_2_ when considering the IAST selectivity, which ranged between 20 and 59 for all benzoxazine samples at 0 ℃, also depicted in Figure 46 [108]. Where high IAST selectivity factors indicate high selectivity towards CO_2_, these results show a clear increase in selectivity from N_2_ [108]. Therefore, benzoxazines would be effective at filtering CO_2_ from flue air streams, although there is still sufficient CH_4_ selectivity. [107] Konnola et al. also obtained high selectivity factors for CO_2_ and N_2_, in the range of 21–28, confirming the excellent separation ability of CO_2_ from blends with N_2_ observed in the two lines plotted in Figure 46 [109].

In addition to determining what factors affect CO_2_ adsorption and selectivity, several studies attempted to fit adsorption to mathematical models or determine the mechanisms occurring. Alhwaige et al. used the Langmuir and Freundlich models, which states that the amount of CO_2_ adsorbed (*q*) relates to the pressure (*p*) through Langmuir adsorption parameters (*a* and *b*) via the following equation [37]:

*q* = *ap*_1_ + *bp* (2)

The Freundlich model was proposed as an alternate, but equally valid, model to relate adsorption and pressure, along with the Freundlich proportionality constant *K_f_* and heterogeneity parameter *n*, where

*q* = *K_f_ p*_1_*n* (3)

Far et al. proposed a general mechanism for the adsorption of CO_2_ in micropores. For benzoxazines that contained pyridinic or pyridonic nitrogen, a carbamate ion was formed endothermically while for benzoxazines containing phenoxide groups, a carbonate product was formed in an energy-neutral process [107]. They also noted that CO_2_ captured as carbonate was continually captured through a cascading effect until micropores had been filled [107].

##### Organic Compounds

**Figure 47:**
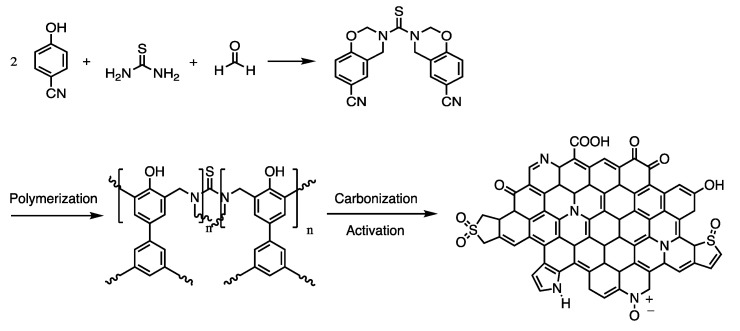
Synthesis scheme for N,S functionalities in the porous carbons (reproduced from the work in [112] with permission).

Polybenzoxazine has been reported as a catalyst carrier [62] and an adsorptive desulfurization [112] material to absorb organic pollutants. Pollutant sequestration is an important research field due to the continuing emission of toxic organic pollutants. For example, organosulfur compounds are prevalent emissions from transportation fuels which largely contribute to acid rain [112]. In addition, such organic pollutants can poison the catalysts that are designed to sequester them by hindering their activity. Zhao et al. prepared a 4-cyanophenol, thiourea, and formaldehyde polybenzoxazine with abundant micropores to help adsorb sulfur compounds in transportation fuels [112] as shown in Figure 47. The authors achieved a high adsorptive capacity of 83.3 mg S/g. 

**Figure 48:**
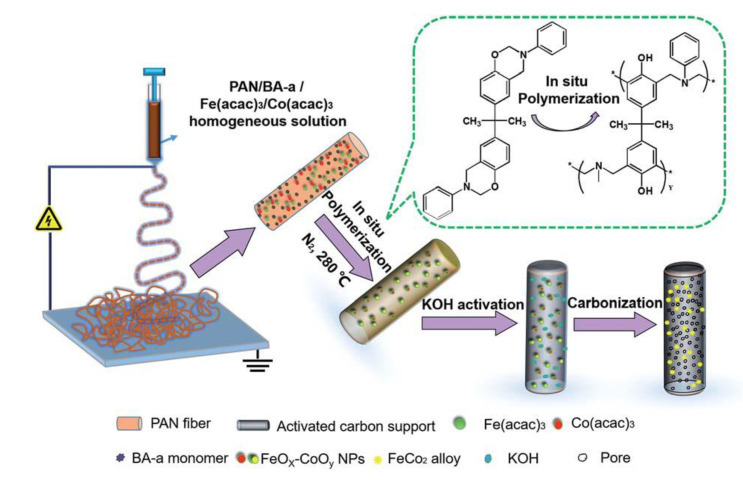
Synthesis scheme of FeCo_2_ crystals supported on N-doped hierarchical structured porous carbon fibers (reproduced from the work in [62] with permission).

Zhu et al. employed polybenzoxazine-derived porous carbon fiber as a catalyst carrier to activate peroxymonosulfate (PMS). Advanced oxidation processes, or AOPs, degrade organic pollutants into lower molecular weight molecules, which then makes them more bioavailable. The authors prepared various molar ratios of Fe_x_CO_2_ alloyed crystals supported on a hierarchical pore polybenzoxazine as shown in Figure 48. The adsorptive and oxidative properties worked together to efficiently remove pollutants [62].

#### 4.2.5. As Non-Metallic Catalysts

Technological development of electric cars, computers, building materials, and batteries has increased the demand for rare earth metals. Because of this, rare-earth metal mining is increasing throughout the world. Unfortunately, metal mines are the cause of disastrous environmental catastrophes, despite the metal’s use in the “clean energy” industry. For example, the Berkeley Pit, a copper mine in Butte, Nevada, has created staggering environmental problems. First, deep shaft mining grew into a network of 10,000 miles, some extending more than a mile beneath the surface. Next, a pit mine was opened which consumed a good part of the city. Once the mining was completed, 13.25 billion pounds of copper in total, the pumps that once removed groundwater from the tunnels and adjacent pit were shut off. As sulfur permeates the bedrock and meets air and water, a caustic compound capable of dissolving almost any metal is produced. 

Today, the mile wide, mile and a half long, and quarter mile deep human-made pit is filled with a growing 28 billion gallons of corrosive, acidic, and toxic water. In addition, the acidic, lead-laden soil is devoid of organic matter, so little vegetation grows in the whole district. For all practical purposes, restoration, remediation, and/or clean up are not considered because it would be extremely expensive. 

The appetite for metals remains unabated despite staggering environmental impacts and impossible remediation strategies. There is a strong research interest in developing metal-free, economic, and environmentally friendly electrocatalyst materials used for a number of reactions [98,113,114,115,116]. Electrocatalytic splitting of water can be achieved by oxygen evolution reaction (OER) (Bawre et al. [113]) and hydrogen evolution reaction (HER) (Thirukumaran et al. [114,115]). Metal-free oxygen reduction reaction (ORR) for fuel cells and metal–air batteries was investigated by Li and Yang [98]. Compared to commercial Pt/C catalysts used in ORR, a material fabricated from triphenylimidazole-containing polybenzoxazine precursors showed higher current density at 0.92V (J_@0.92_, 0.251 vs 0.141 mA/cm^2^). Platinum is an expensive, limited resource which necessitates the need for metal-free catalysts [98].

For the purpose of developing more sustainable and environmentally friendly alternatives to petroleum-based resources, hydrogen can be used as an energy source. Hydrogen can be efficiently and practically obtained from water splitting via HER [114,115]. Polybenzoxazine has been employed as a metal-free, non-toxic, and inexpensive alternative to electrocatalyst materials used for water splitting [114,115]. To date, platinum and its alloys are predominantly used as catalysts for HER, however high cost, low abundance, and the aforementioned environmental problems of metal mining restricts usage [98,114,115]. However, pristine carbon materials are poor catalysts because they are electrochemically inert [115]. Polybenzoxazine’s unique and highly tailorable structure allows for chemical modification by means of heteroatom doping, which enhances wettability among other properties [115]. Heteroatoms, N and O, are present in each chemical repeat unit of polybenzoxazines without purposefully adding these atoms.

**Figure 49:**
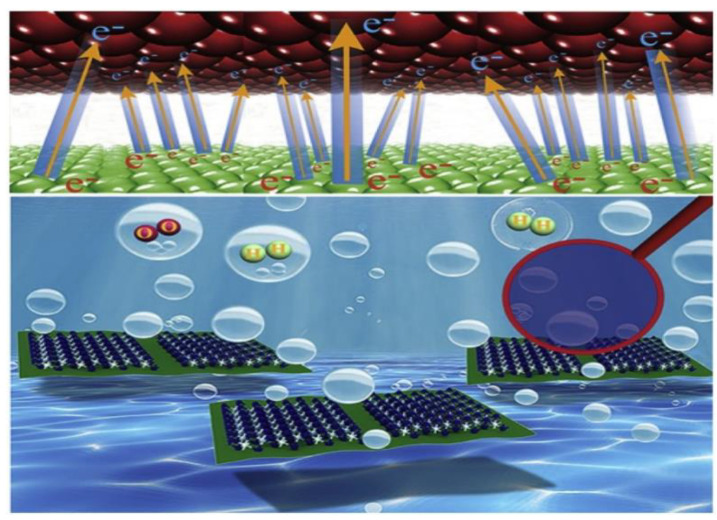
Enhanced photocatalytic reaction at monolithic aerogel interface for stable water splitting reactions (reproduced from the work in [117] with permission).

In addition to hydrogen, monolithic aerogels are an emerging photocatalytic material with potential in the clean energy production industry owing to impressive specific surface area, interconnected open-frameworks, and highly macroscopic operability and recoverability. A branched benzoxazine monomer was employed as an initiator and the resulting material exhibited potential to be utilized in water splitting reactions as illustrated in Figure 49 [117].

Lignocellulosic biomass is a cost-effective, plentiful material with substantial energy potential. Lignocellulosic biomass contains C5 and C6 sugars, key reactants for the production of valuable 16-platform chemicals such as 5-hydroxymethylfurfural, Furfural, levulinic acid, succinic acid, and fumaric acid. These chemicals are crucial intermediates for synthesizing high-value biobased chemicals, polymers, and solvents. Succinic acid is used in a number of industries as a biobased material. It is synthesized from the catalytic hydrogenation of petroleum-based maleic acid, so producing Succinic acid from inedible, biomass-derived compounds would reduce fossil fuel consumption [116].

#### 4.2.6. As Organic Sensors

A prevalent concern of modern industry is the emission of volatile organic compounds (VOCs). VOCs, such as n-hexane or acetone, are hazardous and can cause problems to human health. Some industries utilize VOCs as feedstock or solvents within their process, so it is important to have real-time sensors to detect trace amounts of VOCs. Sensor films have been widely employed as gas sensitive materials to monitor hazardous pollutant species or specific industrial gases. Conductive networks are formed by the connection of filler particles throughout the polymer matrix. Once organic vapors are introduced, the matrix phase swells and the interparticle path of filler gets cut off, increasing the resistance and shorting the conductive circuit. Thubsuang et al. [118] reported high absorption capacity carbon aerogels derived from polybenzoxazine as a suitable filler for gas sensing polymer composites [118]. 

#### 4.2.7. As Carbon Dots

**Figure 50:**
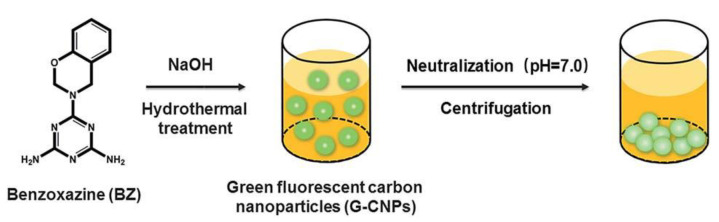
Synthesis schematic of basophilic green fluorescent carbon nanoparticles derived from benzoxazine (reproduced from the work in [119] with permission).

Carbon dots (CDs), also known as carbon quantum dots or carbon nanodots, are emerging as a promising carbon-based photoluminescent nanomaterial [41,42,119]. CDs have been attracting attention since their discovery in 2004 [41,119]. Properties include excellent photoluminescence [42,119], low toxicity [42,119], high aqueous solubility [41,42], high photostability and resistance to photobleaching [41,42], excellent optical properties [41], biocompatibility [42], and low-cost synthesis [42,119]. Such properties allow for CD applications in bio-imaging, photovoltaic devices, photocatalysis, biosensing, drug delivery, and photodynamic therapy [41,42]. For instance, Fang et al. [119] used CDs as fluorescent probes to detect heavy or transition metal ions for the prevention of pollution and protection of human health. The authors used benzoxazine to synthesize basophilic green fluorescent carbon nanoparticles by a one-pot hydrothermal treatment in NaOH aqueous solution, depicted in Figure 50 [119].

**Figure 51:**
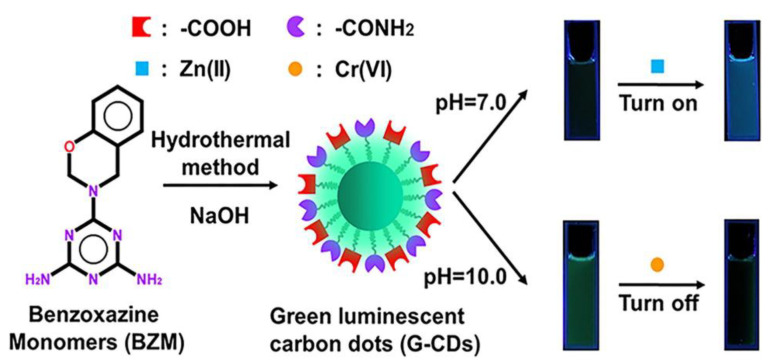
Graphical representation of pH-controlled green luminescent carbon dots with fluorescence turn-on and turn-off detection (reproduced from the work in [41] with permission).

However, CD fluorescence turn-on detection is not the only control mechanism. Another control mechanism is based on fluorescence quenching or recovery [41]. Fang and Lu [41] fabricated a novel multifunctional CD and found that physical properties of CDs, such as solubility, depend on surface functional groups. The high nitrogen content of benzoxazine makes it an excellent raw material to prepare CDs with nitrogen functional groups and a high quantum yield, see Figure 51 [41].

**Figure 52:**
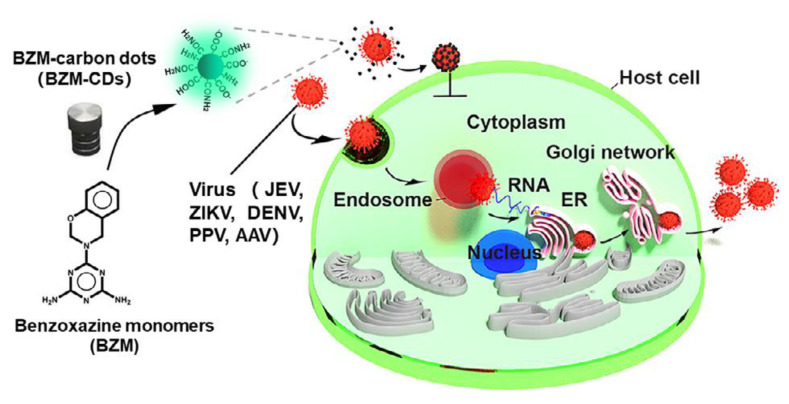
Benzoxazine-derived CD binding to the surface of virions, thus preventing entry of virions into the host cell; the first step of virus–cell interaction (reproduced from the work in [42] with permission).

Another interesting application of CDs derived from benzoxazine is as a safe and effective anti-flaviral agent which exhibits broad-spectrum activity. Roughly 200 million cases of flaviviruses, such as Zika, Japanese encephalitis, and Dengue virus, occur each year. Flaviviruses have no definitive antiviral therapies available. Huang et al. developed a benzoxazine derived CD which is active against both flaviviruses and non-enveloped viruses, suggesting therapeutic potential of such nanoparticles as depicted in Figure 52 [42].

#### 4.2.8. As Electromagnetic Shielding Materials

Electromagnetic interference (EMI) affects electronic device function, information safety, and can cause human health problems. Metals and alloys currently serve as shielding materials because of their high electrical conductivity and developed manufacturing technology, however they are limited by high density, brittleness, and corrosion susceptibility. Conductive polymer composites are emerging as lightweight, anti-corrosive, and inexpensive materials with the ability to meet the advancing EMI demands [120,121]. However, polymers are often limited by inferior thermal properties, and the addition of conductive fillers can harm mechanical characteristics [120]. Among studied fillers, carbon nanoparticles have many advantages such as high conductivity and ease of modification [121]. 

**Figure 53:**
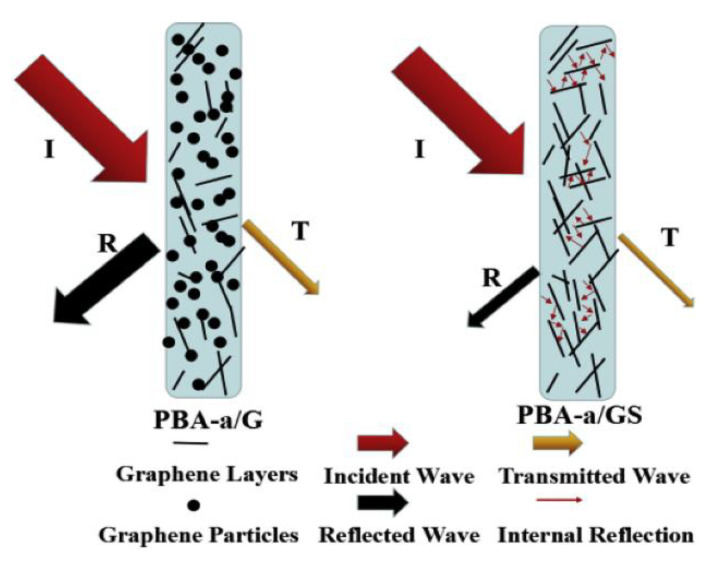
Schematic of EMI shielding of polybenzoxazine/graphene nanocomposite samples PBA-a/G and PBA-a/GS (reproduced from the work in [121] with permission).

Among polybenzoxazine’s numerous advantageous properties, high thermal resistance and stability are two characteristics that researchers exploit [120]. For instance, Zhang et al. employed polybenzoxazine resin and conductive graphene to investigate EMI shielding properties of polybenzoxazine/graphene nanocomposites as shown in Figure 53 [121]. 

#### 4.2.9. As Intumescent Coating Materials

Intumescent coatings are characterized by the formation of a foam char layer when exposed to a heat source. Intumescent coatings are used as a passive, aesthetic fire resistance measure and are often painted onto the exterior of a material. The intumescence phenomena require three components to occur: a carbon source, blowing agent, and acid source [117,122]. As the temperature increases, the acid source esterifies the carbon source, which generates a residue through an incomplete combustion reaction. Consequently, inert gasses will be released. When the blowing agent (commonly melamine) is degraded the char layer will expand [117,122]. 

Benzoxazine has applications in the aerospace industry and within electric circuits due to its intrinsic flame retardancy and high char yield [122,123]. Benzoxazine as an intumescent coating has only been recently reported, and it has not been reported as the main component of an intumescent system. Therefore, Beraldo et al. [122] aimed to develop an efficient benzoxazine-based intumescent coating. Two important parameters to determine fire resistance of a material are limiting oxygen index (LOI) and UL-94 flame test. LOI is a theoretically calculated fire parameter; a material with a LOI above 28 is considered to be self-extinguishing. The UL-94 flame test is another critical fire parameter and V-0 is the best achievable rating. Liu and colleagues [123] reported the LOI of poly(PH-ddm) increased from 31.1 to 37.9 and the UL-94 rating improved from V-1 to V-0 after the addition of PHB-apa. 

#### 4.2.10. As Recoverable Porous Magnetic Carbons

**Figure 54:**
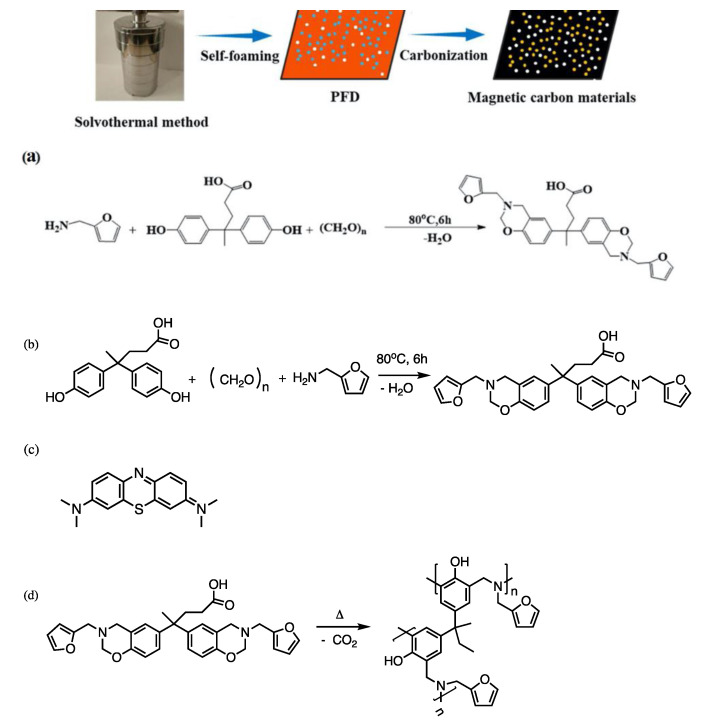
(**a**) Preparation process of novel biobased benzoxazine synthesized from DPA and furfurylamine with Fe_3_CO_4_ nanoparticles dispersed prepared by the solvothermal method with simultaneous polymerization and self-foaming reactions of benzoxazine monomer and thermal decomposition of Fe(acac)_2_; (**b**) Synthesis of the benzoxazine monomer; (**c**) the structure of the dye used, and (**d**) the polymerization scheme of the benzoxazine monomer. Only idealized structure is shown. (reproduced from the work in [124] with permission).

The adsorption of environmental pollution, such as toxic dyes from industrial effluent, is an important research topic. Magnetic separation is a common adsorption technique which also overcomes problems associated with conventional powdery adsorbents. Magnetic adsorbents are typically composed of magnetic nanoparticles dispersed throughout the matrices. Porous carbons are good candidates as adsorbates due to their large surface area, controllable morphologies, and abundant functional surface groups. Feng et al. published a report on a novel fully biobased benzoxazine which was synthesized from diphenolic acid (4,4’-bis(4-hydroxyphenyl) (DPA) and furfurylamine (2-(aminomethyl) as shown in Figure 54. They studied dye absorption performance, magnetic separability, and recyclability of the magnetic carbon material [124].

#### 4.2.11. As Carbon Monoliths/Adsorbents

**Figure 55:**
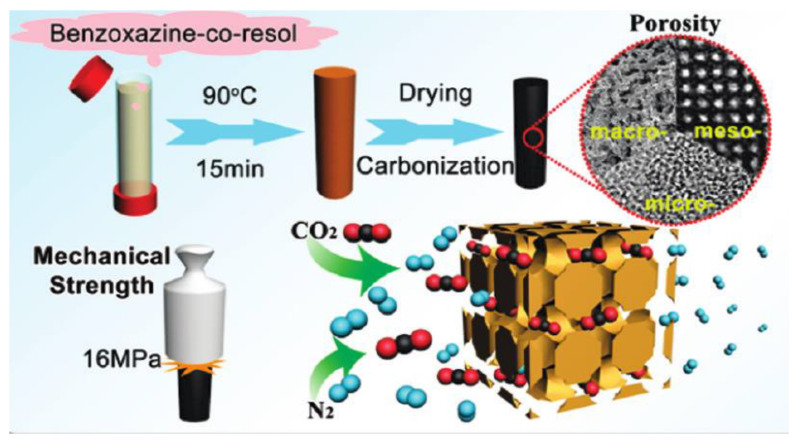
Benzoxazine-co-resol based porous carbon monoliths for high-performance CO_2_ capture (reproduced from the work in [125] with permission).

Selective adsorption of CO_2_ is essential for air pollution remediation. Specifically, CO_2_ capture from power plant effluent is a current research focus. In addition, CO_2_ can be utilized as a renewable carbon source. Numerous porous carbon materials have been developed for CO_2_ capture such as zeolites, amine-modified silicas, and new classes of hybrid crystalline solids. Commercially available activated carbons have a high surface area in the range of 1000–3100 m^2^/g and abundant microporosity (0.4–1.4 cm^3^/g). These porous carbons perform well at high pressures, but the CO_2_ adsorption capacity lessens at low relative pressures. In addition, most carbonaceous CO_2_ sorbents are a powder consisting of fine carbon particles, which yields disadvantages such as high pressure drop, low heat and mass transfer, and mechanical attrition [125,126]. Porous carbons with high surface area, good chemical resistance, and excellent thermal stability are most suitable for various CO_2_ capture conditions as depicted in Figure 55. One strategy to improve CO_2_ sorption at low pressure is to introduce amine groups. Amine groups can be introduced by post modification techniques or by copolymerization with an amine containing precursor. However, a disadvantage of amine-modified sorbents is that they require high pressure and/or high temperature gradients to achieve complete desorption [125]. 

It is attractive to develop an efficient synthesis procedure for monolithic porous carbons. Compared to carbon powders, carbon monoliths exhibit controllable structure, good mechanical properties, high permeability, good regeneration abilities at ambient temperatures and pressures, and high interconnectivity. Research groups who have reported the design and fabrication of such porous carbons include Hao et al. [125] and Sevilla et al. [126]. For example, Sevilla et al. reported the synthesis process of a carbon monolith that contains a graphitic framework. The graphitic framework is a way to overcome common carbon monolith drawbacks such as poor electronic conductivity and amorphous framework. Hao et al. prepared nitrogen containing porous carbon monoliths with high mechanical strength through the self-assembly of poly(benzoxazine-co-resol) followed by a carbonization process [125].

## 5. Extreme Property Materials

**Figure 56:**
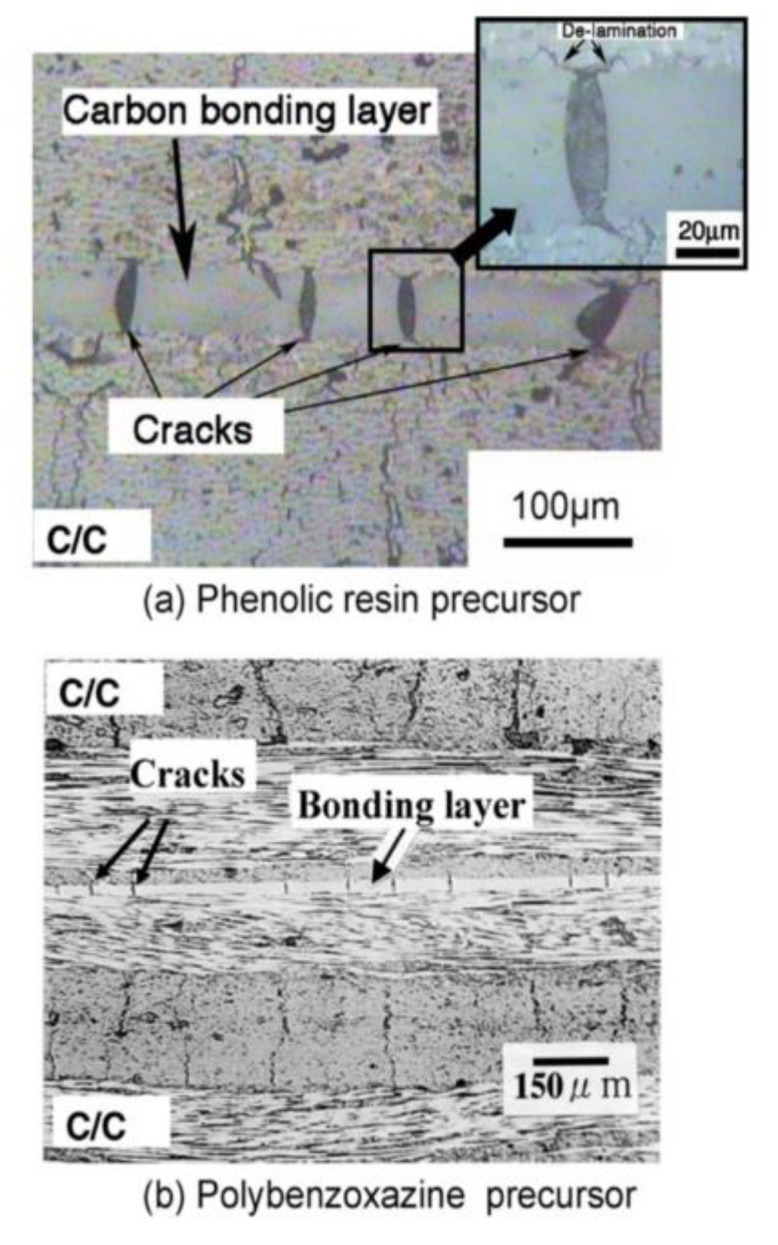
Optical photomicrographs of bonding layer cross sections derived from phenolic resin hot-pressed at 1 MPa during carbonization (**a**) and polybenzoxazine at 10 MPa (**b**) (reproduced from the work in [128] with permission).

Carbon/carbon composites (C/Cs) have many advantageous properties including good electrical conductivity and fire resistance [127] as well as low density and high strength [128]. For these reasons, C/Cs have applications in high temperature environments, such as aerospace [128] and lithium-ion battery systems [127]. However, some unprotected C/Cs undergo thermo-oxidative degradation at temperatures as low as 500 °C, which leads to load-bearing failures from the weakening mechanical properties [127,128]. Bonding techniques at high temperatures have been reported by many authors, but the effects of processing parameters were not discussed in detail. Therefore, Hatta et al. [128] studied optimum processing conditions for carbon bonding at room temperature. An important conclusion is that carbon bonding strength is influenced by the thickness of the bonding layer. Figure 56 depicts micro-images of cross sections of the carbon bonding layer for a phenolic resin (a) and polybenzoxazine (b) [128]. 

As stated earlier, mechanical property reduction of unprotected C/C composites is caused by thermo-oxidative degradation, which occurs at temperatures as low as 500 °C [127]. Thermo-oxidative decomposition is accelerated by matrix cracking, so effective oxidation-protective coatings have been studied by Jin and Ishida and reported that the combination of inorganic filler and high char yielding benzoxazines exhibited the char yield of the coating as high as 86% under oxidative environment of air. A non-coated C/C composite disintegrated under air at 700 °C in less than 10 h, whereas properties were sustained for as long as 1000 h with the newly developed coating [127]. 

Structural aircraft materials also need protection from lasers. Traditional laser protection materials fail at a certain temperature, but polybenzoxazines’ high thermal stability and char yield make it a promising material for laser protective coatings [129]. Xu et al. [129] studied laser protective coatings, while Ma et al. [130] studied laser ablation behavior on polybenzoxazine-based composites. Laser protective coatings are an important design component for aircraft materials. Polybenzoxazines high char yield makes it a promising ablation material. The authors studied the ablation behavior of various polybenzoxazine-based composites through high-intensity continuous laser irradiation tests [129] and analyzed the high energy laser-induced damage mechanisms [130].

## 6. Conclusions

Polybenzoxazines are extremely effective for numerous advanced applications when prepared as carbon monoliths, aerogels, xerogels, nanofibers, and nanospheres. Benzoxazines can be synthesized in various efficient (energetically, cost, and environmentally) ways, including through the use of green solvents (e.g., water) or solventless methods. They can also be carbonized, and the identified degradation products released in this process and amount of crosslinking are key to determining the possible carbonization mechanisms, which includes dimerization from the free radical products. Synthesis and carbonization of polybenzoxazines can be carefully controlled to yield products with desired properties and as a result, benzoxazines are highly suitable for a number of advanced applications. Note that the following applications and qualities can be enhanced by the incorporation of heteroatoms, namely, nitrogen and oxygen, but also boron, phosphorus, and sulfur among others.

Two prominent benzoxazine materials are films and mechanically-sound foams (with specific regards to a high strength and compressive modulus). These properties are also consistent in polybenzoxazine aerogels and xerogels. Manufacturing nanospheres and ferrous nanofibers can uniquely yield highly porous materials, particularly with regards to high micropore and mesopore volumes. KOH activation can be used to increase pore characteristics, along with solvent selection, electrospinning, silica loading, or controlled reaction parameters (such as monomer concentration).

Other advanced applications of polybenzoxazines include electrodes and adsorbents, in addition to catalysts, organic sensors, carbon dots, electromagnetic shielding materials, intumescent coatings, recoverable porous magnetic carbon, and carbon monoliths. As electrodes, activated carbon materials are effective as both EDLCs and Faradic pseudo-capacitors, as well as materials in batteries as a sulfur host to improve sulfur utilization and lifetime. Benzoxazines make excellent adsorbents for gaseous CO_2_ and are effective at selecting CO_2_ from airstreams containing other gases, thus reducing air pollution from carbon emissions. On an additional environmental note, the high-tailorability of benzoxazines that allows them to be used as organic catalysts is key to preventing the extraction of fossil fuels as a catalyst source. The physical reaction of polybenzoxazines to volatile organic compounds also makes them effective organic sensors for environmental purposes, and their magnetic adsorption properties allow benzoxazines to be used for organic dye removal. Carbon dots, another application of benzoxazines, have several unique properties including photoluminescence, and can be used as antiviral agents, among other uses. As electromagnetic shielding materials, polybenzoxazines have particularly high thermal properties, that along with their conductivity, gives them good electromagnetic interference capabilities. Thermal properties, especially char yield, are further effective for polybenzoxazines to be used as intumescent coatings. Benzoxazines, when prepared as composites, also exhibit particularly high thermal and electrical properties and are thus ideal candidates for extreme applications, such as aerospace applications.

## Figures and Tables

**Table 1:** Conductivity, electrochemical properties, and CO_2_ uptakes at 1 bar of NPCs [95].

		Cg (F/g)		Cg/SBET (µF/cm^2^)		CO_2_ Uptake (mmol/g)	
Sample	(S/cm^−1^)	1 A/g	40 A/g	1 A/g	40 A/g	0 °C	25 °C
NPC-c	2.96	69.6	-	54.7	-	2.60	1.79
NPC-0	3.07	303	128	24.6	10.4	5.47	3.65
NPC-1	3.39	338	182	27.0	14.5	6.20	3.95
NPC-2	3.44	362	200	17.8	9.80	5.11	3.38
NPC-3	3.58	286	184	12.6	8.12	4.15	2.96

## Data Availability

For all relevant data, contact the publishers of the referenced articles.
